# Range Estimation of Snapping Shrimp Sounds Using TDOA Structures and a Multilayer Perceptron

**DOI:** 10.3390/s26206386

**Published:** 2026-10-09

**Authors:** Yonghwa Choi, Hansoo Kim, Sunhyo Kim, Bum-Kyu Kim, Donhyug Kang, Sungho Cho, Gihoon Byun

**Affiliations:** 1 Korea Institute of Ocean Science and Technology, Busan 49111, Republic of Korea; yhchoi@kiost.ac.kr (Y.C.); hskim@kiost.ac.kr (H.K.); sunhyo@kiost.ac.kr (S.K.); kimbk91@kiost.ac.kr (B.-K.K.); dhkang@kiost.ac.kr (D.K.); 2 University of Science and Technology, Busan 49111, Republic of Korea; 3 Department of Convergence Study on the Ocean Science and Technology, Korea Maritime and Ocean University, Busan 49112, Republic of Korea; gbyun@kmou.ac.kr

**Keywords:** range estimation, snapping shrimp, time-difference of arrival, multilayer perceptron, SAVEX15, frequency-difference matched field processing, steered frequency-wavenumber analysis

## Abstract

This study proposes a TDOA-MLP method for estimating the range of snapping shrimp sources from the array-wide time-difference-of-arrival (TDOA) structure measured by a vertical line array. BELLHOP arrival-time replicas were generated for the SAVEX15 environment by varying source range, array tilt, and maximum water depth, and a multilayer perceptron was trained to estimate source range with array tilt as an auxiliary output. To provide a more stringent assessment of range generalization, six continuous source-range intervals were excluded from training, and only their inner portions were used for blocked-holdout evaluation, providing an approximately 2-m separation from the nearest training ranges. The resulting overall range-estimation mean absolute error (MAE) and root mean square error (RMSE) were 0.864 and 1.056 m, respectively, with regional MAEs of 0.762, 0.728, and 1.119 m for the near-, mid-, and far-range regions. Additional analyses showed that the estimation error increased with controlled arrival-time perturbations and varied with the maximum-depth condition. When applied to experimental SAVEX15 snapping shrimp data, the TDOA-MLP produced range distributions and snap-to-snap variations generally consistent with those obtained using template matching, steered frequency-wavenumber analysis, and frequency-difference matched-field processing. Relative to template matching, the mean absolute relative range difference was 4.54%. Because ground-truth source ranges were unavailable for the experimental data, the field results demonstrate consistency among the methods rather than absolute localization accuracy. The proposed method provides a low-dimensional learning-based approach for estimating the range of impulsive snapping shrimp signals from array-wide TDOA structures.

## 1. Introduction

Snapping shrimp are prominent biological sound sources in warm, shallow-water environments, continuously producing intense, impulsive broadband signals [1,2,3]. A snapping sound is generated by the collapse of a cavitation bubble formed during the rapid closure of the shrimp’s asymmetrically enlarged claw and is characterized by a high sound pressure level and a broad frequency bandwidth over a short duration [4,5]. In areas inhabited by large aggregations of snapping shrimp, the accumulation of individual snaps produces a persistent crackling sound that constitutes a major component of high-frequency ambient noise [1,2,3]. Although snapping shrimp sounds can interfere with underwater acoustic communication and sonar systems, they can also serve as naturally occurring acoustic illuminators or sources of opportunity for ocean acoustic applications [3,4,6]. Accurate localization of individual snaps is essential for using snapping shrimp sounds in passive acoustic sensing [6,7]. Knowledge of snap locations can support the characterization of shrimp aggregation patterns, biological activity around marine structures, habitat monitoring, and the analysis of seabed and environmental conditions [6,7]. Previous studies have localized snapping shrimp sounds using time differences of arrival (TDOA), wavefront curvature, the delay between direct and surface-reflected arrivals, or multipath structure observed across an array [6,7]. When a source is located in the near field of the array, the received wavefront is curved rather than planar, and the resulting TDOA structure contains range information in addition to source bearing [6,7]. In practice, however, the deployment of large-aperture arrays can be difficult, and with a small-aperture array, range estimation for distant sources may remain limited even when bearing can still be resolved [7].

To address this limitation, a geometry-based method was proposed to estimate snapping-shrimp range from the TDOA between the direct and surface-reflected arrivals using a small-aperture array [7]. By treating the sea surface as an acoustic reflector, the method enables range estimation for far-field snaps. In real data, however, multiple snaps often occur within a short time and exhibit similar acoustic characteristics, making it difficult to associate each direct arrival with its corresponding surface-reflected arrival [7]. In addition, uncertainties in receiver depth and orientation, sea-surface conditions, and water depth can affect the resulting range estimates [7].

Snapping shrimp sounds recorded during Shallow-Water Acoustic Variability Experiment 2015 (SAVEX15) have been used for source localization and array-calibration studies with a vertical line array (VLA) [6,8]. The experiment was conducted in approximately 100-m-deep shallow water, where snapping shrimp sounds were continuously recorded on a bottom-moored, 16-element VLA with an aperture of approximately 56 m [6,8]. A previous study exploited the assumption that snapping shrimp are located near the seabed to localize individual snaps in the range–tilt domain and demonstrated that the same signals could be used to calibrate VLA tilt [6]. To process high-frequency snapping shrimp signals received on a sparse VLA, steered frequency–wavenumber analysis (SFK) was subsequently proposed [8]. Adaptive SFK was later developed by incorporating adaptive processing techniques, including the minimum-variance distortionless response and a white-noise-gain constraint, to improve the performance of conventional Bartlett-based SFK [9]. These studies demonstrated the feasibility of estimating source range and array tilt from SAVEX15 snapping shrimp data. However, they rely on physics-based replica or ambiguity-surface calculations and remain subject to mismatch between the assumed and actual environments [6,8,9]. In contrast to these previous SAVEX15 studies, which estimate source location through physics-based replica matching or frequency–wavenumber processing, the present study formulates the problem as supervised regression from an array-wide TDOA vector to source range and array tilt.

Time difference of arrival is a widely used feature for passive source localization [10,11]. TDOA-based localization estimates the geometric relationship between a source and an array from the relative arrival times measured across the sensors and, compared with time-of-arrival methods, places less stringent requirements on absolute time synchronization between the source and receivers [10,11]. In realistic underwater environments, however, localization performance can be affected by uncertainty in the sound-speed structure, sensor-position errors, changes in array orientation, multipath propagation, and errors in arrival-time estimation [10,11]. Previous studies have therefore sought to improve TDOA-based localization by combining TDOA and frequency difference of arrival, compensating for sensor-position errors, estimating an unknown sound speed, and exploiting multipath arrivals through virtual sensors [10,11]. TDOA-based approaches are also widely used for the passive acoustic localization and tracking of marine animals, where signal association becomes particularly important when multiple sources or acoustically similar signals are present simultaneously [12].

Recently, machine-learning- and deep-learning-based approaches have been actively investigated to learn the nonlinear relationship between measured acoustic features and source location [13,14,15,16,17,18,19]. Matched field processing (MFP) is a representative physics-based method that estimates source location by generating replica fields at candidate positions using an acoustic propagation model and comparing them with the measured field [13,14]. Its performance, however, is sensitive to the accuracy of environmental information, including the sound-speed structure, seabed properties, and water depth, and can degrade when the assumed environment differs from the actual ocean conditions [13,14,15,17]. As an alternative, underwater source localization has been formulated as a supervised classification or regression problem [13,14,19]. Proposed input features include normalized sample covariance matrices, frequency-domain acoustic pressure, eigenvector-based features, and spectrograms, which have been used to train models such as feedforward neural networks, support vector machines, random forests, generalized regression neural networks, kernel extreme learning machines, and convolutional neural networks [13,14,15,16,17,18,19].

However, most previous machine-learning-based studies of underwater source localization have relied on acoustic-field or frequency-domain features, and only limited attention has been given to directly using the arrival-time structure of short impulsive signals such as snapping shrimp sounds [13,14,15,16,17,18,19]. Because an individual snap exhibits a distinct arrival structure over a short duration, the intersensor arrival-time differences can be represented by a relatively low-dimensional TDOA vector [1,2,3,4,5,6,7,8]. This TDOA structure reflects changes in relative arrival time associated with source range and array geometry, consistent with established TDOA-based localization principles. In this study, this low-dimensional representation is used as the input feature for learning the relationship between the arrival-time structure and the source range of impulsive snapping shrimp signals [6,7,8].

This study proposes a multilayer perceptron (MLP)-based method, referred to as TDOA-MLP, for estimating the range of snapping shrimp sounds from the array-wide TDOA structure measured on a vertical line array. The proposed method uses a 15-dimensional TDOA vector referenced to the bottom hydrophone as input and estimates source range as the primary output together with array tilt as an auxiliary output. The training data consist of BELLHOP arrival-time replicas generated for the SAVEX15 environment. The trained model is then applied to experimental SAVEX15 snapping shrimp data and compared with template matching, steered frequency–wavenumber analysis, and frequency-difference matched field processing.

The proposed method uses a low-dimensional TDOA vector to jointly estimate the source range and array tilt of each snap. Its performance, however, may depend on the fidelity of the propagation-model-based training data and the accuracy of arrival-time extraction from real measurements.

The main contributions of this study are summarized as follows. First, established TDOA-based geometric information is formulated as a low-dimensional array-wide input representation for learning-based range estimation of impulsive snapping shrimp signals received on the SAVEX15 VLA. Second, a TDOA training dataset is generated as a function of source range, array tilt, and water-depth condition using BELLHOP arrival-time replicas, and the TDOA-MLP is developed based on this dataset. Third, a multi-output learning framework is introduced in which source range is treated as the primary output and array tilt as an auxiliary output, allowing tilt-induced variations in the TDOA structure to be incorporated into the learned mapping. Fourth, the proposed method is applied to experimental SAVEX15 snapping shrimp data, and its applicability and limitations are examined through comparisons with template matching, SFK, and frequency-difference matched field processing (FDMFP) [20,21].

The remainder of this paper is organized as follows. Section 2 describes the proposed TDOA-MLP and the comparison methods, including SFK and FDMFP. Section 3 presents the procedure for generating the BELLHOP-based replica dataset and constructing the TDOA structure. Section 4 describes the MLP input and output, network architecture, training strategy, and template-matching baseline. Section 5 summarizes the SAVEX15 experimental environment and the extraction of TDOAs from real snapping shrimp signals. Section 6 presents the range-estimation results for the simulation and real-data cases and compares them with those obtained using the existing methods. Section 7 discusses the physical interpretation of TDOA-based learning, the effects of array tilt and environmental mismatch, and the applicability and limitations of the proposed method. Finally, Section 8 presents the conclusions.

## 2. Range Estimation Methods

This section describes the methods used to estimate the range of snapping shrimp sounds. The proposed method, TDOA-MLP, uses the TDOA structure of snapping shrimp signals received on a vertical line array as its input feature. To compare the proposed method with existing physics-based approaches, steered frequency–wavenumber analysis (SFK) and frequency-difference matched field processing (FDMFP), both of which have previously been applied to snapping shrimp localization in SAVEX15, are considered [8,9]. TDOA-MLP is a regression-based approach that learns the mapping between a TDOA vector and the corresponding range–tilt labels, whereas SFK and FDMFP are physics-based methods that compare modeled responses over candidate source locations.

### 2.1. Proposed TDOA-MLP

This study proposes TDOA-MLP, a method that estimates the range of snapping shrimp sounds from the arrival-time structure measured on a vertical line array. Because a snapping shrimp snap is an intense impulsive signal of short duration, its arrival time can be identified on each array channel. When the source lies in the near field of the array, the received wavefront is curved rather than planar. Consequently, the intersensor arrival-time differences contain geometric information related to both source range and array tilt.

**Figure 1:**
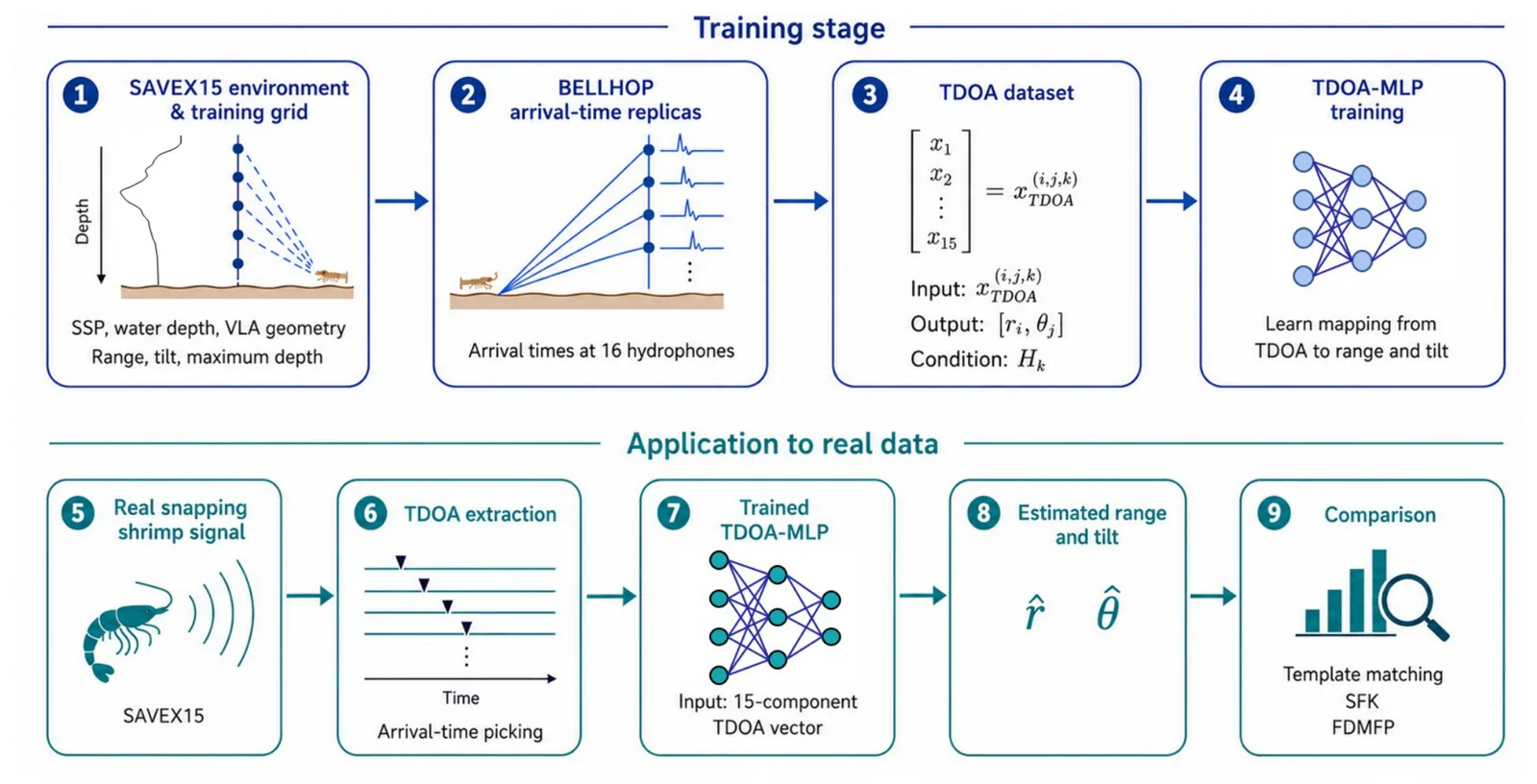
Overall framework of the proposed TDOA-MLP for snapping shrimp range estimation.

Figure 1 illustrates the overall processing flow of the proposed TDOA-MLP. During the training stage, BELLHOP arrival-time replicas are generated for the SAVEX15 environment, and the MLP is trained using 15-dimensional TDOA vectors referenced to sensor 16 together with their corresponding range–tilt labels. During the application stage, a TDOA vector of the same form is constructed from each real snapping shrimp snap, and the trained model is used to estimate the source range and array tilt. The generation of the replica dataset and construction of the TDOA structure are described in Section 3, while the MLP architecture and training procedure are presented in Section 4.

### 2.2. Steered Frequency-Wavenumber Analysis

Frequency–wavenumber analysis transforms the time–space signals received on an array into the frequency–wavenumber (ω–*k*) domain, allowing the frequency content and direction of arrival to be analyzed simultaneously [22,23]. When a broadband signal is received, striations appear in the ω–*k* spectrum, and their slopes are related to the signal arrival direction [22,23,24,25]. However, when the signal frequency exceeds the design frequency of the array, spatial aliasing can occur. The striations may then shift or fold along the wavenumber axis, making it difficult to effectively exploit the full frequency band [24,25].

The SAVEX15 VLA is a sparse array consisting of 16 sensors with an intersensor spacing of 3.75 m, whereas the dominant frequency content of snapping shrimp sounds extends into the kilohertz range. Direct application of conventional ω–*k* analysis can therefore suffer from degraded localization performance due to spatial aliasing. To mitigate this problem, SFK compensates the received signals using a steering vector or replica for each candidate source location before performing the ω–*k* analysis [8]. When the received signals are compensated using the replica for an assumed candidate location, the striation in the ω–*k* spectrum becomes vertically aligned near k=0 when that candidate coincides with the true source location.

In SFK, the ambiguity-surface value for a candidate location (r,z) is obtained by integrating, over the frequency band, the k=0 component of the steered ω–*k* spectrum generated after replica compensation. Conceptually, this can be expressed as follows:

(1) $$P_{\mathrm{SFK}}\left(r,z\right)=\int_{\omega_{L}}^{\omega_{H}}\tilde{S}\left(r,z,k=0,\omega \right),d\omega ,$$

Here, $\tilde{S}\left(r,z,k=0,\omega \right)$ denotes the steered ω–*k* spectrum obtained after compensating the received signals with the replica for the candidate location (r,z), and $\omega_{L}$ and $\omega_{H}$ are the lower and upper bounds of the analyzed frequency band, respectively. The location that maximizes $P_{\mathrm{SFK}}\left(r,z\right)$ is interpreted as the candidate whose replica best matches the received signal. The physical consistency of the localization result can also be assessed by confirming that the striation in the corresponding ω–*k* spectrum is vertically aligned near k=0.

In previous studies using SAVEX15 snapping shrimp data, BELLHOP-based replicas were employed instead of simple plane-wave steering vectors because the snapping shrimp signals were generated by high-frequency, near-field sources located close to the array [8]. Because array tilt strongly affects localization performance, the results were also examined in the range–tilt domain by varying the candidate tilt [8,9]. In the present study, SFK is used as a physics-based comparison method for TDOA-MLP. Range estimates are obtained using replicas calculated for the same SAVEX15 environment and applied to the real snapping shrimp signals.

### 2.3. Frequency-Difference Matched Field Processing

FDMFP is a localization method that forms low-frequency difference components from pairs of high-frequency signal components and performs matched field processing in the resulting difference-frequency band [20,21,26]. The method originates from the frequency-difference beamforming concept proposed by Abadi et al. [26]. Frequency-difference beamforming selects two frequency components from a broadband signal and uses their product to perform beamforming at a difference frequency lower than the original signal band. Therefore, even when the array is spatially sparse relative to the original signal frequency and conventional beamforming is strongly affected by spatial aliasing, the lower effective frequencies in the difference-frequency band can improve the robustness of array processing [26].

Worthmann et al. extended the frequency-difference concept to conventional, or Bartlett, matched field processing and proposed FDMFP [20]. Conventional MFP calculates replica fields for candidate source locations using an acoustic propagation model and compares them with the measured array data to construct an ambiguity surface. In shallow water, however, direct processing of high-frequency signals can produce large phase discrepancies between the measured field and the replica because of mismatch between the actual and modeled environments. Because this environmental mismatch generally becomes more severe with increasing frequency and propagation range, it is a major factor limiting the localization performance of conventional MFP at high frequencies [20]. FDMFP seeks to reduce this sensitivity by performing matched field processing with low difference-frequency components generated from pairs of high-frequency components, rather than directly matching the original high-frequency signals [20].

The principal input used in FDMFP is the autoproduct. Let $P_{j}\left(\omega \right)$ denote the frequency-domain signal measured at the *j*-th sensor. The autoproduct for center frequency ω and difference frequency Δω is defined as follows:

(2) $${\mathrm{AP}}_{j}\left(\omega ,\Delta \omega \right)=P_{j}\left(\omega +\frac{\Delta \omega }{2}\right)P_{j}^{*}\left(\omega -\frac{\Delta \omega }{2}\right),$$

Here, ${\left(\cdot \right)}^{*}$ denotes complex conjugation. Because the autoproduct is formed from the phase difference between two nearby high-frequency components, its phase variation is governed by the difference frequency Δω, rather than by the original signal frequency ω. Thus, even though no low-frequency signal is physically transmitted, ${\mathrm{AP}}_{j}\left(\omega ,\Delta \omega \right)$ can be interpreted as a pseudo-field resembling the low-frequency acoustic field that would be formed at the difference frequency Δω [20,21].

In FDMFP, the autoproducts are used to construct a cross-spectral density matrix (CSDM) in the difference-frequency band. The autoproduct vector for the entire array is defined as

(3) $$\mathrm{AP}\left(\omega ,\Delta \omega \right)={\left[{\mathrm{AP}}_{1}\left(\omega ,\Delta \omega \right),{\mathrm{AP}}_{2}\left(\omega ,\Delta \omega \right),\ldots ,{\mathrm{AP}}_{N}\left(\omega ,\Delta \omega \right)\right]}^{T},$$

The difference-frequency CSDM can then be defined as follows:

(4) $$K_{\mathrm{FD}}\left(\omega ,\Delta \omega \right)=\frac{\mathrm{AP}\left(\omega ,\Delta \omega \right){\mathrm{AP}}^{H}\left(\omega ,\Delta \omega \right)}{{\mathrm{AP}}^{H}\left(\omega ,\Delta \omega \right)\mathrm{AP}\left(\omega ,\Delta \omega \right)},$$

Here, ${\left(\cdot \right)}^{H}$ denotes the Hermitian transpose. The ambiguity surface is then computed by matching $K_{\mathrm{FD}}\left(\omega ,\Delta \omega \right)$ with the replica vector $w_{\mathrm{FD}}\left(x,\Delta \omega \right)$, calculated at the difference frequency Δω for a candidate location x. Using a Bartlett processor, the FDMFP ambiguity surface can be expressed as follows:

(5) $$P_{\mathrm{FD}}\left(x,\omega ,\Delta \omega \right)=w_{\mathrm{FD}}^{H}\left(x,\Delta \omega \right)K_{\mathrm{FD}}\left(\omega ,\Delta \omega \right)w_{\mathrm{FD}}\left(x,\Delta \omega \right),$$

For a broadband signal, the final FDMFP output is obtained by incoherently averaging the ambiguity surfaces calculated over multiple center frequencies $\omega_{m}$ and difference frequencies $\Delta \omega_{n}$ within the signal band, rather than using a single frequency pair. This can be expressed as follows:

(6) $$P_{\mathrm{FD},\mathrm{avg}}\left(x\right)=\frac{1}{N_{\Delta }}\sum_{n=1}^{N_{\Delta }}\frac{1}{N_{\omega }}\sum_{m=1}^{N_{\omega }}P_{\mathrm{FD}}\left(x,\omega_{m},\Delta \omega_{n}\right),$$

Here, $N_{\omega }$ is the number of center frequencies used in the averaging, and $N_{\Delta }$ is the number of difference frequencies. The candidate location that maximizes $P_{\mathrm{FD},\mathrm{avg}}\left(x\right)$ is interpreted as the location at which the measured autoproduct field best matches the difference-frequency replica.

The principal advantage of FDMFP is that it constructs relatively low-frequency difference-field information from high-frequency received signals, thereby mitigating the environmental mismatch and sparse-array limitations that strongly affect conventional high-frequency MFP [20]. In addition, normalization of the autoproduct and the magnitude-squared operation suppress the influence of the source waveform, allowing localization without precise knowledge of the transmitted signal [20,21]. However, because FDMFP is a nonlinear processing method based on autoproducts, multipath environments generate not only the desired self-terms but also undesired cross-terms. These cross-terms can reduce the dynamic range of the ambiguity surface or increase sidelobe levels. Moreover, lowering the difference frequency improves robustness to environmental mismatch but reduces spatial resolution, resulting in an inherent trade-off [20,21].

Subsequent studies on adaptive FDMFP extended Bartlett FDMFP to adaptive processors such as the minimum-variance distortionless response (MVDR), minimum-covariance-matrix (MCM), multiple-signal classification (MUSIC), and minimum-mean-power (MMP) processors and evaluated their performance under noisy conditions [21]. These studies showed that adaptive methods can improve the peak-to-sidelobe ratio or dynamic range under certain conditions. However, because of the nonlinear nature of the autoproduct, the resolution improvement typically expected from adaptive processing in conventional MFP is not always achieved [21]. Accordingly, the present study uses the Bartlett-based FDMFP employed in previous SAVEX15 snapping shrimp localization studies as a comparison method and examines the consistency of the TDOA-MLP range estimates with those obtained using the conventional physics-based difference-frequency approach.

## 3. Replica Dataset Generation

This section describes the procedure used to generate the replica dataset for training the TDOA-MLP. Because accurate information on the source range of snapping shrimp and the array tilt is difficult to obtain directly in an at-sea environment, arrival-time replicas were generated using an acoustic propagation model for various source ranges and array-tilt conditions. The relative arrival-time differences with respect to a reference sensor were then calculated from the generated replicas and organized into TDOA structures for MLP training. The training dataset was constructed based on the SAVEX15 environment by varying the source range, array tilt, and maximum water depth.

### 3.1. BELLHOP Arrival-Time Replicas

BELLHOP, a ray-based acoustic propagation model, was used to generate the replica dataset [27]. BELLHOP is a high-frequency approximation model that calculates the arrival structure between a source and receivers in a given ocean environment, including ray paths, arrival times, amplitudes, and arrival angles. In this study, the TDOA replicas were constructed using the direct-path arrival time at each channel among the arrivals predicted by BELLHOP.

The BELLHOP calculations incorporated the sound-speed structure, water depth, VLA geometry and sensor positions, source depth, and seabed acoustic parameters of the SAVEX15 experimental site. Snapping shrimp were assumed to be biological sound sources located near the seabed, and the source depth was set to 0.02 m above the seabed for each maximum-depth condition. This setting assumes that the center of the shrimp is located approximately half its body length above the seabed, considering a representative body length of approximately 0.04 m [5]. This source height was adopted as a representative modeling assumption rather than an exact description of the position of every snapping shrimp, and possible variability in source height relative to the local seabed was not explicitly included in the present training dataset. The receiving array was modeled as a 16-element vertical line array with an interelement spacing of 3.75 m. The uppermost sensor was defined as Channel 1, and the lowermost sensor as Channel 16.

**Figure 2:**
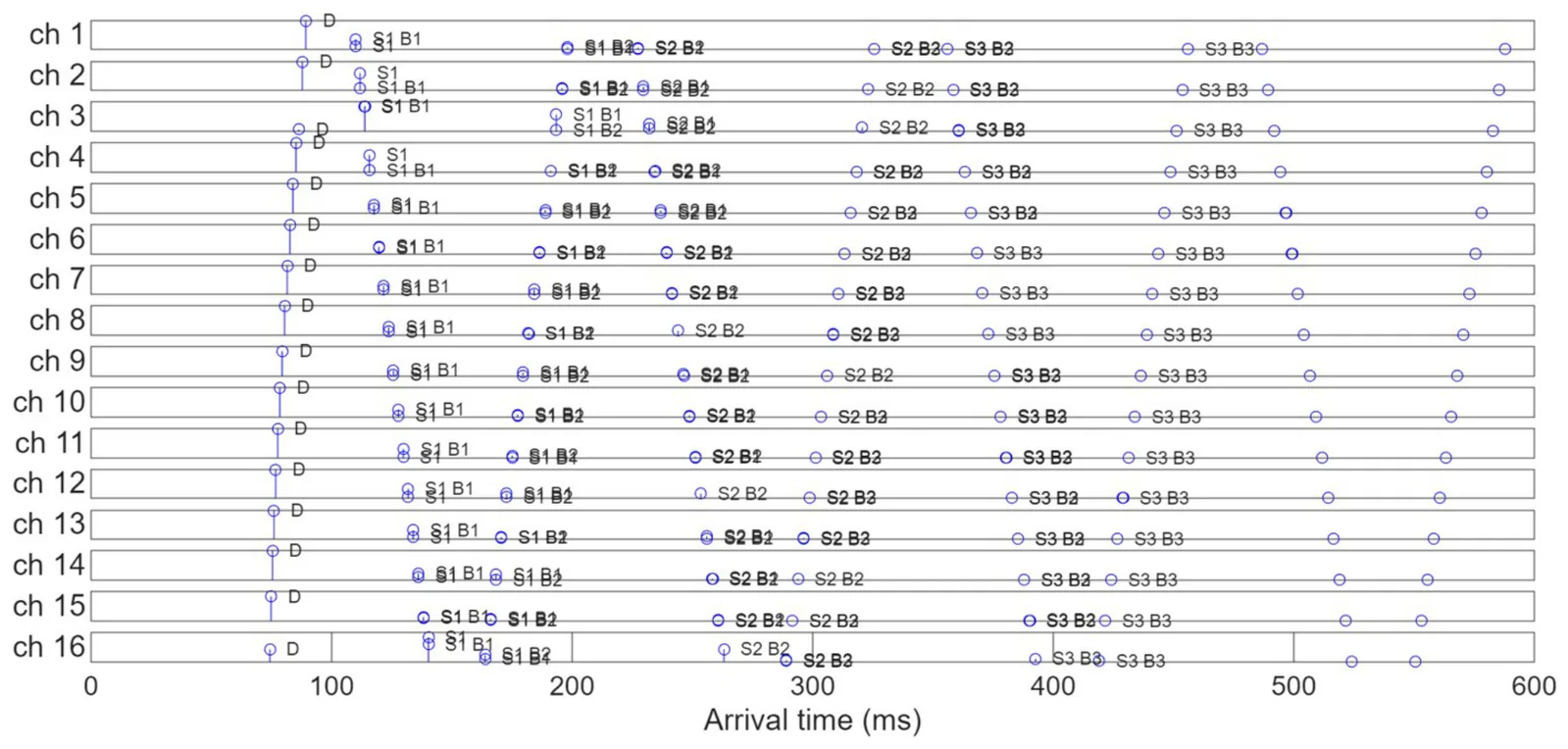
Example of BELLHOP arrival-time replicas for a snapping shrimp source at 110 m range, 100 m water depth, and 99.98 m source depth.

Figure 2 presents an example of the BELLHOP arrival-time replicas calculated for a representative condition. The example shows the arrival-time structure across the 16 receiving channels for a source range of 110 m, a maximum water depth of 100 m, and a source depth of 99.98 m. Although multiple arrivals can be predicted at each channel, only the arrival time corresponding to the direct path was used to construct the TDOA structure in this study. This procedure was repeated for all combinations of source range, array tilt, and maximum water depth to generate the TDOA training dataset.

### 3.2. Training Parameters

The training data for the TDOA-MLP were generated by varying the source range, array tilt, and maximum depth. The source range was defined as the horizontal distance from the VLA to the source located near the seafloor and was varied from 10 to 200 m at 0.01 m intervals. The 0.01 m interval was used to densely sample the continuous range-dependent TDOA manifold for dataset generation and does not imply a physical range resolution of 0.01 m. To reduce the influence of closely spaced samples during model training and evaluation, the training set was randomly subsampled and the model was evaluated using continuous blocked-holdout intervals separated from the nearest training ranges, as described in Section 4. The maximum-depth conditions were determined based on the measured local bathymetry around the VLA and were selected to represent the observed depth variability in the study area. Within approximately 200 m of the VLA, the water depth varies gradually around 100 m; therefore, four maximum-depth conditions of 99, 100, 101, and 102 m were used to reflect this observed depth variability in the training dataset. The array tilt was varied from −4° to 4° at 0.1° intervals relative to the vertical orientation. Although the snapping shrimp data analyzed in this study were collected at a different time, previous studies using the same VLA during SAVEX15 reported that the array tilt estimated from ship-radiated noise varied within approximately ±4°. Accordingly, the training range for the array tilt was set to −4° to 4° based on these experimental observations [28,29].

**Figure 3:**
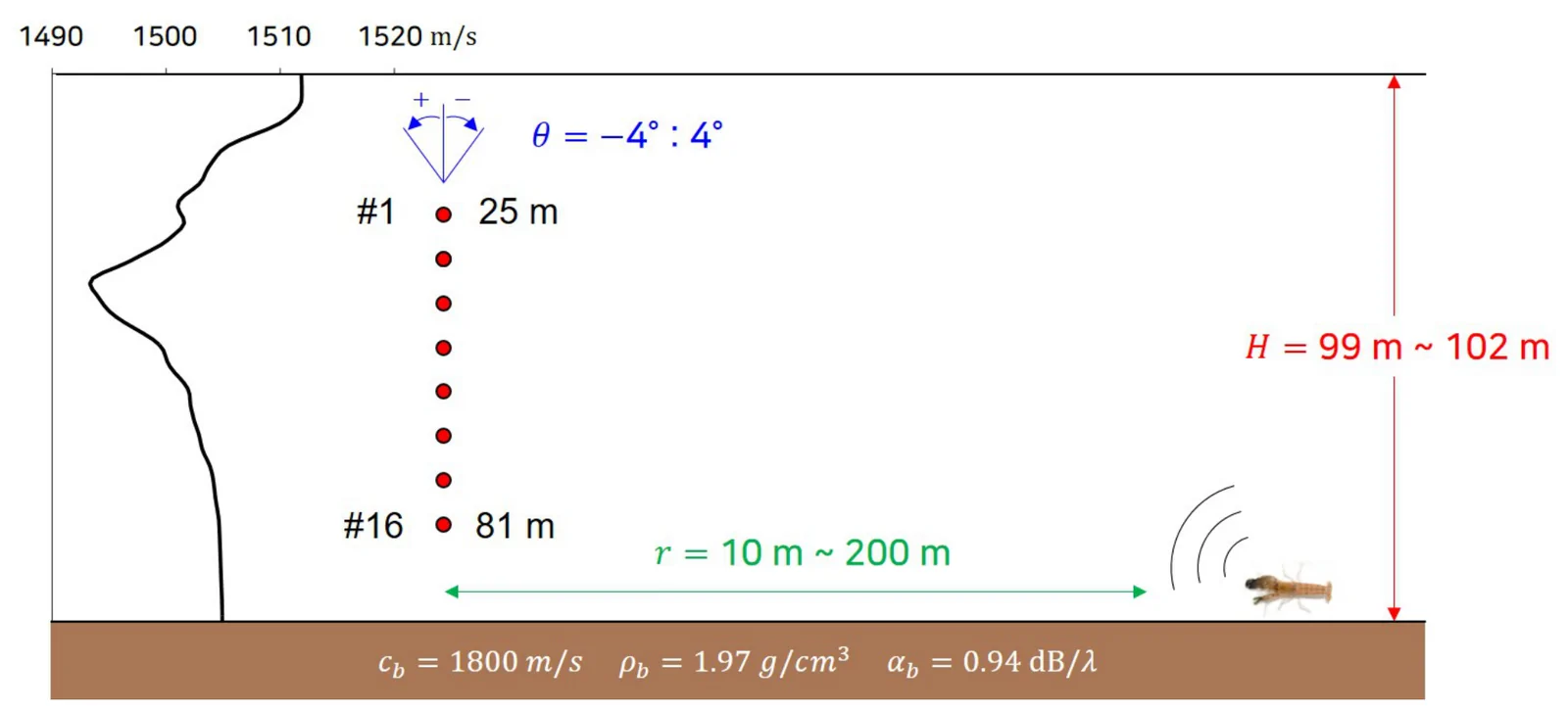
Geometry and parameter ranges used for BELLHOP-based TDOA replica dataset generation. The source was positioned 0.02 m above the seafloor, with the source range varied from 10 to 200 m, array tilt from −4° to 4°, and maximum depth from 99 to 102 m.

Figure 3 schematically illustrates the source–receiver array geometry used to generate the BELLHOP-based TDOA replica dataset. The snapping shrimp source was positioned close to the seafloor, and the arrival time at each hydrophone was calculated while varying the VLA tilt and the horizontal source range. The resulting array-wide TDOA structures corresponding to the different environmental conditions were then used as the training data for the TDOA-MLP.

The parameters used to generate the BELLHOP-based TDOA replica dataset are summarized in Table 1.

Here, the maximum depth is not an output estimated by the model but is used solely as an environmental condition for generating the replica dataset. Specifically, the output labels of the TDOA-MLP are the source range and array tilt, whereas the maximum depth is used as an environmental parameter in replica generation to incorporate water-depth-dependent variations in the arrival-time structure into the training data. In this way, the local depth variability around the VLA is represented in the replica dataset.

The seabed acoustic properties were fixed rather than varied as training environmental parameters. Because the input to the proposed method consists of relative differences among the selected direct-path arrival times, variations in the seabed acoustic properties were not included in the training parameter space. However, the effects of bottom-property variations on arrival selection and the resulting TDOA structure were not evaluated separately in this study and should be investigated in future work.

### 3.3. TDOA Structure

For each BELLHOP simulation condition, the arrival times at the 16 receiving channels were obtained, and the relative arrival-time differences were calculated using the lowermost sensor, Channel 16, as the reference. Let $t_{i}$ denote the selected arrival time at the *i*-th sensor and $t_{16}$ the arrival time at the reference sensor. The TDOA for the *i*-th sensor is then defined as follows:

(7) $$\Delta t_{i}=t_{i}-t_{16},\;i=1,2,\ldots ,15,$$

Accordingly, the TDOA vector for a given source location and array-tilt condition is constructed as follows:

(8) $$x_{\mathrm{TDOA}}={\left[\Delta t_{1},\Delta t_{2},\ldots ,\Delta t_{15}\right]}^{T},$$

This TDOA vector represents the relative arrival-time structure of the remaining 15 sensors with respect to the reference sensor, Channel 16. As the source range changes, the curvature of the wavefront incident on the vertical array also changes, resulting in variations in the relative arrival-time differences among the sensors. In addition, changes in the array tilt alter the actual positions of the sensors and therefore modify the TDOA structure even at the same source range. Thus, $x_{\mathrm{TDOA}}$ can be regarded as a low-dimensional feature that simultaneously contains information on the source range and array tilt.

The complete replica dataset can be expressed in terms of the range index *i*, array-tilt index *j*, and maximum-depth-condition index *k* as follows:

(9) $$x_{\mathrm{TDOA}}^{\left(i,j,k\right)}⟶{\left[r_{i},\theta_{j}\right]}^{T},$$

Here, $r_{i}$ denotes the *i*-th source-range condition, $\theta_{j}$ denotes the *j*-th array-tilt condition, and $H_{k}$ denotes the *k*-th maximum-depth condition. Although $H_{k}$ is used as an environmental condition for generating the TDOA structure, it is not included in the MLP output labels. Thus, the TDOA-MLP is designed to partially account for TDOA variability associated with changes in water depth by learning TDOA structures generated under multiple maximum-depth conditions.

The resulting TDOA dataset is used as the input to the MLP, while the corresponding source range $r_{i}$ and array tilt $\theta_{j}$ are used as the output labels.

## 4. Multilayer Perceptron

### 4.1. Input and Output

The input to the TDOA-MLP is the 15-dimensional TDOA vector defined in Equation (8). The input TDOAs were initially calculated in seconds and then converted to milliseconds. Subsequently, each TDOA component was standardized using the mean and standard deviation calculated from the training set to reduce differences in scale among the input features and improve numerical stability during training.

(10) $$\tilde{x}=\frac{x_{\mathrm{TDOA}}-\mu_{x}}{\sigma_{x}},$$

Here, $\tilde{x}$ denotes the standardized TDOA input vector, while $\mu_{x}$ and $\sigma_{x}$ denote the vectors of the mean and standard deviation, respectively, calculated for each TDOA component from the training set. The same standardization parameters determined from the training set were applied to the validation set, holdout simulation data, and experimental data.

The MLP outputs consist of the source range and array tilt. The primary quantity of interest in this study is the source range *r*, whereas the array tilt θ is jointly estimated as an auxiliary output to account for changes in the TDOA structure caused by variations in the array orientation. Accordingly, the target vector is defined as follows:

(11) $$y={\left[r,\theta \right]}^{T},$$

Because the source range and array tilt have different units and numerical ranges, each output component was standardized using the mean and standard deviation calculated from the training set so that both outputs contributed on comparable scales to the loss calculation.

(12) $$\tilde{y}=\frac{y-\mu_{y}}{\sigma_{y}},$$

Here, $\tilde{y}$ denotes the standardized target vector, while $\mu_{y}$ and $\sigma_{y}$ denote the vectors of the mean and standard deviation, respectively, calculated from the output labels in the training set. Because the trained MLP outputs the standardized source range and array tilt, inverse standardization was applied during inference to recover the estimates in their original physical units.

(13) $$\hat{y}=\hat{\tilde{y}}⊙\sigma_{y}+\mu_{y}={\left[\hat{r},\hat{\theta }\right]}^{T},$$

Here, ⊙ denotes element-wise multiplication, while $\hat{r}$ and $\hat{\theta }$ denote the estimated source range and array tilt, respectively.

### 4.2. MLP Architecture

The TDOA-MLP used in this study is a fully connected multi-output regression model based on a multilayer perceptron, taking a 15-dimensional TDOA vector as input and producing a two-dimensional output vector consisting of source range and array tilt. Because the input dimensionality is relatively small and the relationship between the TDOA structure and the source range and array tilt can be represented as a continuous nonlinear mapping, a multilayer perceptron architecture was adopted without convolutional or recurrent layers.

The TDOA-MLP consists of an input layer, three hidden layers, and an output layer. The three hidden layers contain 128, 128, and 64 nodes, respectively, and a rectified linear unit (ReLU) activation function is applied to each hidden layer. The output layer consists of two nodes to simultaneously estimate the source range and array tilt. Because both outputs are continuous-valued variables, a linear activation function was applied to the output layer. The network architecture used in this study is summarized in Table 2.

The network architecture can be summarized as follows:

(14) $${\tilde{x}}_{\mathrm{TDOA}}\in R^{15}\to \mathrm{FC}\left(128\right)\to \mathrm{ReLU}\to \mathrm{FC}\left(128\right)\to \mathrm{ReLU}\to \mathrm{FC}\left(64\right)\to \mathrm{ReLU}\to \mathrm{FC}\left(2\right)\to \hat{\tilde{y}},$$

Here, $\hat{\tilde{y}}$ denotes the predicted vector of the standardized source range and array tilt. During inference, inverse standardization is applied to recover the prediction as ${\left[\hat{r},\hat{\theta }\right]}^{T}$. This architecture was designed to represent the nonlinear variations in the 15-dimensional TDOA input associated with source range and array tilt while maintaining a relatively simple network structure.

### 4.3. Training Strategy

The TDOA-MLP was trained in a supervised manner using the combined BELLHOP-based replica datasets generated for maximum-depth conditions of 99–102 m. To evaluate range generalization under a more stringent condition, a blocked-holdout strategy was adopted instead of excluding isolated range points. Six contiguous 10-m range intervals, 25–35, 55–65, 85–95, 115–125, 145–155, and 175–185 m, were completely excluded from model training. All array-tilt and maximum-depth samples corresponding to these excluded range intervals were removed from the training set.

For evaluation, only the inner 6-m portions of the excluded intervals, namely 27–33, 57–63, 87–93, 117–123, 147–153, and 177–183 m, were used as the blocked-holdout test set. This configuration provided an approximately 2-m guard interval between each evaluation block and the nearest training range, with a minimum train–test range separation of approximately 2.01 m on the 0.01-m range grid. Thus, the blocked-holdout evaluation reduces the possibility that the test performance is dominated by interpolation from immediately adjacent training samples.

Given the very large number of available training samples, up to 1,500,000 samples were randomly selected without replacement from the remaining training ranges. All array-tilt conditions from −4° to 4° and all four maximum-depth conditions were retained for the selected training ranges.

The training-set-based standardization defined in Section 4.1 was applied to both the inputs and outputs. The training objective was defined as the squared-error loss for the standardized source-range and array-tilt outputs. For a mini-batch containing $N_{b}$ samples, the loss function can be expressed as follows:

(15) $$L=\frac{1}{2N_{b}}\sum_{n=1}^{N_{b}}{∥{\hat{\tilde{y}}}_{n}-{\tilde{y}}_{n}∥}_{2}^{2},$$

Here, ${\tilde{y}}_{n}$ denotes the standardized target vector for the *n*th sample, and ${\hat{\tilde{y}}}_{n}$ denotes the corresponding MLP prediction. By standardizing the source range and array tilt separately and training them jointly under the same squared-error objective, the effects of their different units and numerical scales on the optimization were reduced.

The Adaptive Moment Estimation (Adam) optimizer was used for network training, with an initial learning rate of $10^{-3}$ and a mini-batch size of 4096. The L2 regularization parameter α for the network weights was set to $10^{-5}$. Early stopping was applied using 10% of the training data as an internal validation set. Training was terminated when the validation performance failed to improve by more than the specified tolerance of $10^{-6}$ for 20 consecutive validation checks. The training hyperparameters and data-splitting settings used for the TDOA-MLP are summarized in Table 3.

After training, the trained MLP and the input and output scalers were saved together. For both the holdout simulation and the application to real data, the input TDOA vectors were transformed using the same input scaler before prediction, and the inverse transformation of the output scaler was then applied to recover the estimated source range and array tilt in their original physical units. Because the MLP output layer was formulated as a linear regression layer, some predictions near the boundaries could slightly exceed the parameter domain used for training. Because such outputs correspond to extrapolation beyond the range defined by the training data, the estimated source range and array tilt were constrained after inference to the predefined estimation domains of 10–200 m and $-4^{\circ }$–$4^{\circ }$, respectively. For the 216 experimental snap events, four range estimates and four tilt estimates were located at the imposed boundary values after post-processing. These boundary estimates may reflect either predictions near the edge of the modeled parameter domain or extrapolation beyond the domain prior to the output constraint.

### 4.4. Baseline Method: Template Matching

Template matching was used as a baseline for comparison with and interpretation of the TDOA-MLP estimates obtained from the experimental data. Template matching is a nearest-replica search method that directly calculates the distance between an input TDOA vector and each TDOA vector in the BELLHOP-based replica dataset and then selects the source range and array tilt of the replica with the smallest distance as the estimates.

Although the TDOA-MLP and template matching use the same BELLHOP replica dataset generated for maximum-depth conditions of 99–102 m, they differ in how the dataset is used. The TDOA-MLP learns a nonlinear mapping between the replica TDOA vectors and their corresponding source-range and array-tilt labels, thereby producing continuous regression outputs. In contrast, template matching does not use a learned mapping; instead, it directly searches the entire replica grid for each input sample and selects the nearest discrete condition.

Let $x_{\mathrm{obs}}$ denote the observed TDOA vector and $x_{\mathrm{TDOA}}^{\left(i,j,k\right)}$ the replica TDOA vector generated for source range $r_{i}$, array tilt $\theta_{j}$, and maximum depth $H_{k}$. The optimal replica index for template matching is then defined as follows:

(16) $$\left(i^{*},j^{*},k^{*}\right)=\underset{i,j,k}{\arg\min}{∥x_{\mathrm{obs}}-x_{\mathrm{TDOA}}^{\left(i,j,k\right)}∥}_{2}^{2},$$

The same TDOA unit conversion was applied to both the observed data and the replicas, whereas the StandardScaler-based standardization used for the MLP input was not applied when calculating the template-matching distance. Thus, Equation (16) represents the squared Euclidean distance calculated in the original 15-dimensional TDOA space. The search ranges were 10–200 m for source range and $-4^{\circ }$–$4^{\circ }$ for array tilt, and all replicas generated for maximum depths of 99, 100, 101, and 102 m were included in the search.

(17) $${\hat{r}}_{\mathrm{TM}}=r_{i^{*}},\;{\hat{\theta }}_{\mathrm{TM}}=\theta_{j^{*}},$$

The selected $H_{k^{*}}$ represents the maximum-depth condition of the replica that yields the minimum distance. However, in the primary comparisons of this study, only the source range and array tilt were used as the template-matching estimates.

Template matching was employed as a nearest-replica baseline based on the same BELLHOP TDOA manifold. Because it does not provide the ground truth for the actual source location, its results for the experimental data were interpreted only as a reference for assessing consistency with the TDOA-MLP estimates.

## 5. SAVEX15

This section describes the SAVEX15 experimental environment and received data used to apply the proposed TDOA-MLP to real snapping shrimp signals. During SAVEX15, snapping shrimp signals were continuously recorded using a vertical line array (VLA) deployed in a shallow-water environment with a water depth of approximately 100 m [30]. In this study, data segments in which snapping shrimp signals were clearly observed were selected, and a TDOA vector was constructed for each snap by extracting the arrival time at each hydrophone.

### 5.1. Experimental Environment

The SAVEX15 experiment was conducted in a shallow-water environment with a water depth of approximately 100 m. The receiving array used in this study was a bottom-moored VLA consisting of 16 hydrophones with an interelement spacing of 3.75 m. The uppermost and lowermost hydrophones were located at depths of approximately 25 and 81 m, respectively. The lowermost hydrophone, Channel 16, was used as the reference sensor for the TDOA calculations.

**Figure 4:**
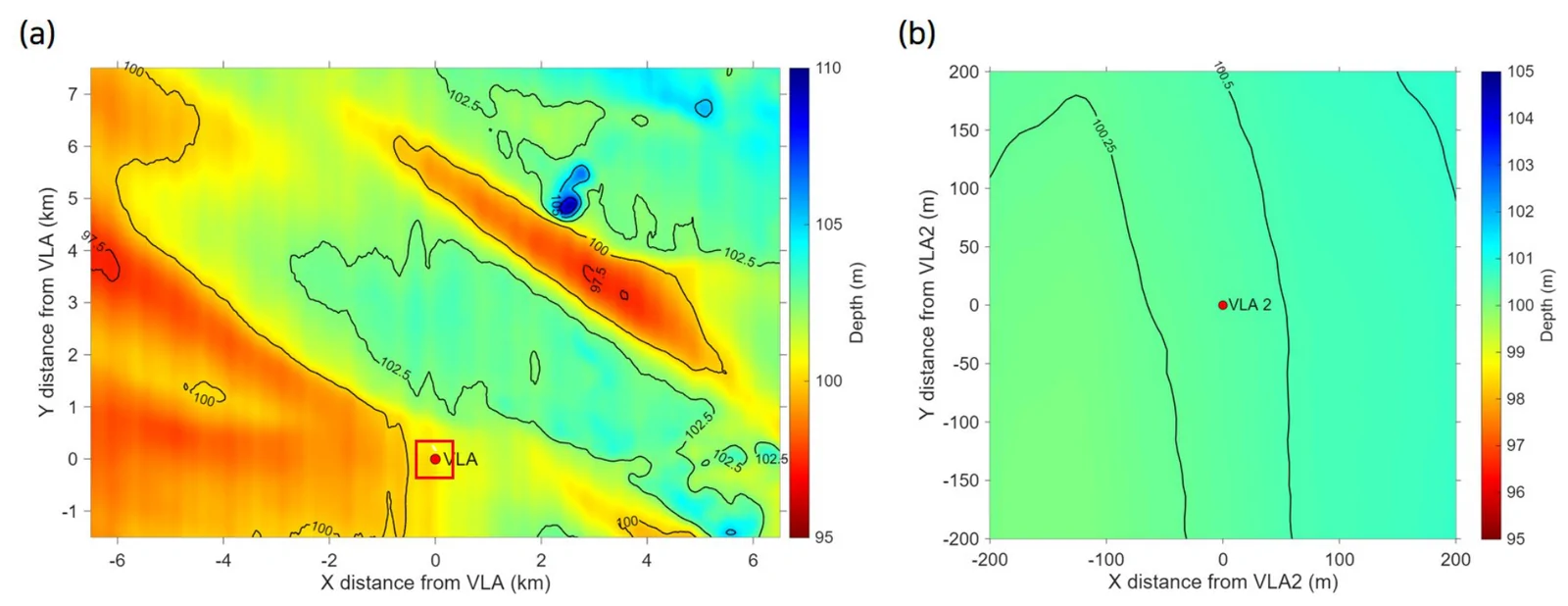
Bathymetric environment of the SAVEX15 experimental site used for BELLHOP-based TDOA replica generation: (**a**) regional bathymetry around the VLA, where the red box denotes the area enlarged in (**b**), and (**b**) local bathymetry within approximately 200 m of the VLA. The red marker indicates the VLA location.

Figure 4 shows the bathymetry around the VLA in the SAVEX15 experimental area. Figure 4a presents the seafloor topography over a relatively broad area centered on the VLA, and the red box indicates the region enlarged in Figure 4b. Figure 4b shows the local bathymetry within approximately 200 m of the VLA, where the water depth varies gradually around 100 m. Based on these measured bathymetric data, the range of maximum-depth conditions used to generate the BELLHOP-based TDOA replica dataset was determined. The sound-speed structure and VLA geometry from the SAVEX15 experimental environment were used in the BELLHOP calculations, and the acoustic parameters of the seabed were set to $c_{b}=1800$ m/s, $\rho_{b}=1.97$ g/cm^3^, and $\alpha_{b}=0.94$ dB/λ.

### 5.2. Snapping Shrimp Signal

In the SAVEX15 data, numerous snapping shrimp signals were received continuously over time. A snapping shrimp snap is an impulsive broadband signal with a high sound pressure level over a short duration. These characteristics produce measurable arrival-time differences across the VLA channels and facilitate the construction of the TDOA structure.

**Figure 5:**
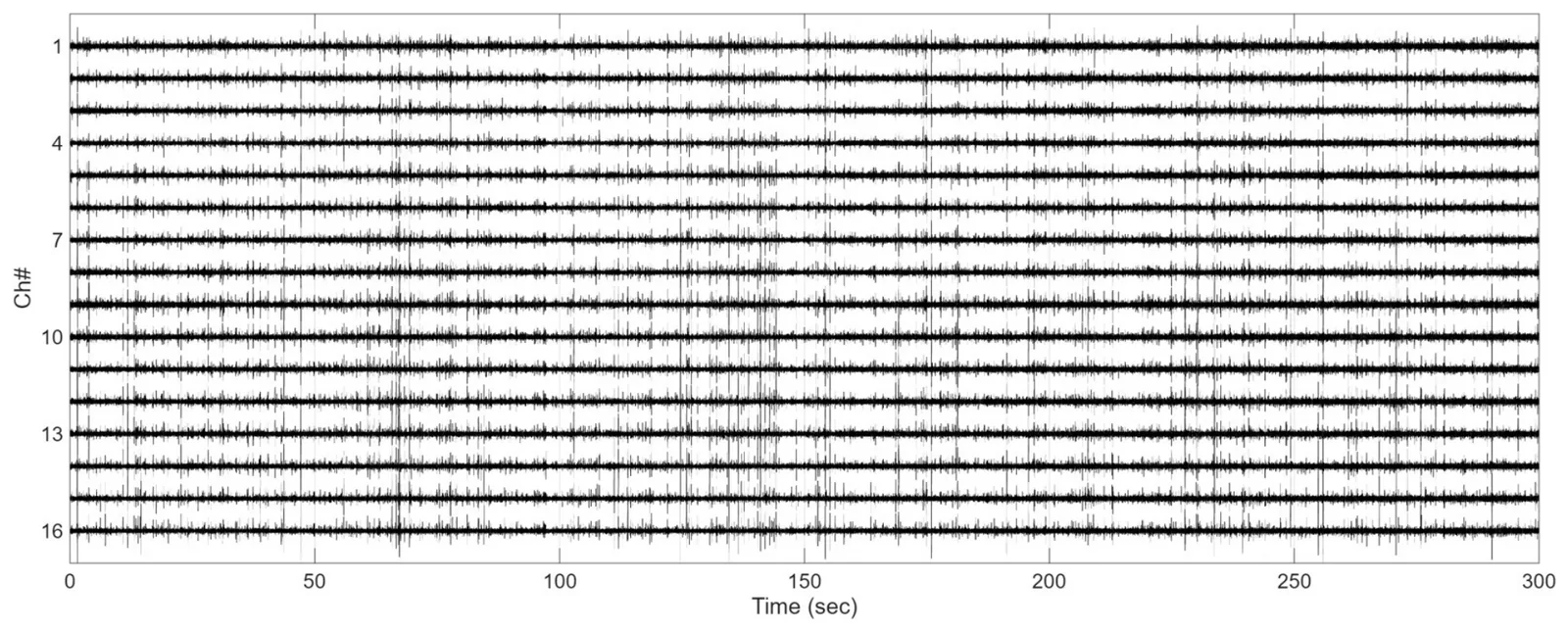
Overview of real snapping shrimp data received on the 16-channel vertical line array during a 5-min interval from 16:51:00 to 16:56:00 on JD 146. Numerous impulsive snapping signals are continuously observed throughout the analysis interval; a representative event and its arrival-time extraction are shown in Figure 6.

**Figure 6:**
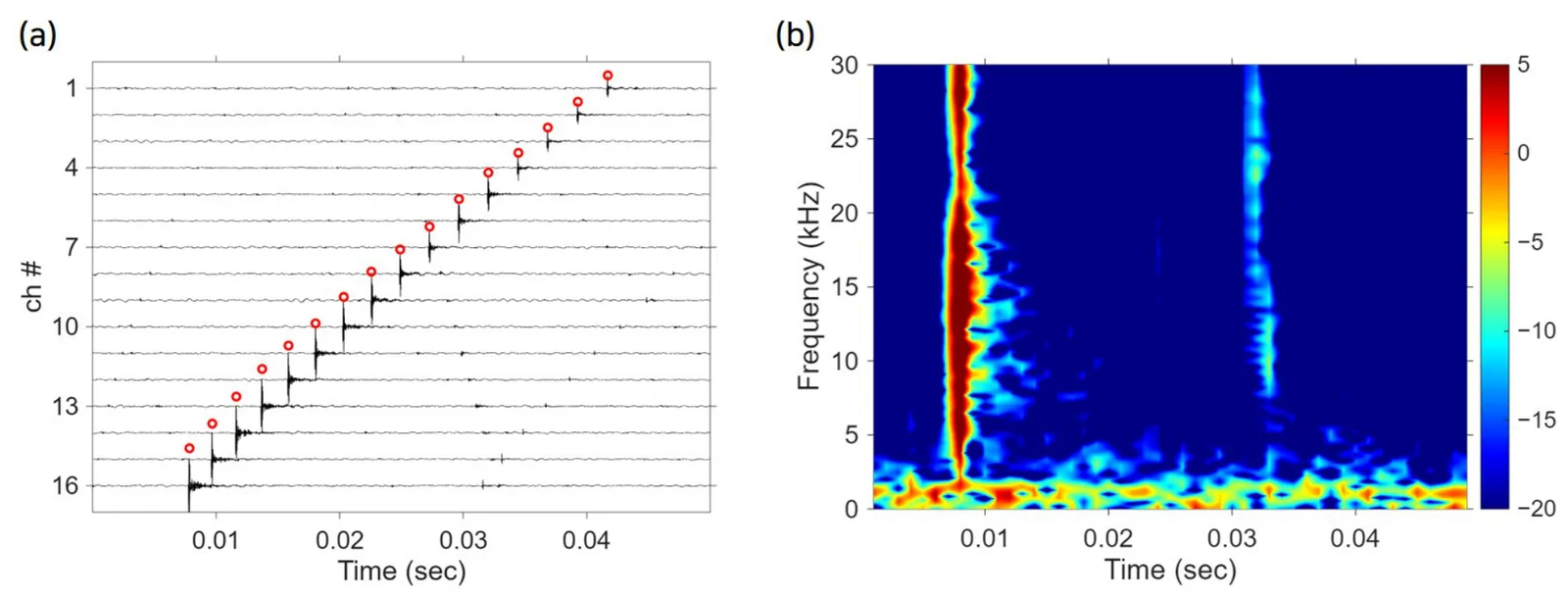
Arrival-time extraction from a real snapping shrimp signal: (**a**) normalized multichannel waveforms received on the vertical line array, where red circles indicate the selected arrival times used for TDOA calculation, and (**b**) spectrogram of the corresponding snap signal received on Channel 16.

Figure 5 shows an overview of the experimental snapping shrimp data used in this study. The data span an approximately 5-min interval from 16:51:00 to 16:56:00 on JD 146 and present the time-domain waveforms received on all 16 channels. Numerous impulsive snapping signals occur continuously throughout the analysis interval. A representative snap event and the corresponding channel-wise arrival-time extraction are shown in detail in Figure 6.

Because snapping shrimp signals have a short temporal duration, an isolated snap event appears as an impulsive signal with a channel-dependent time shift. Individual snap events were subsequently screened and processed as described in Section 5.3.

### 5.3. TDOA

The TDOA extraction procedure for the experimental snapping shrimp signals is illustrated in Figure 6. Figure 6 shows a representative snap event observed during the 1.49–1.54 s interval of the VLA data recorded at 16:51:00 on JD 146. The left panel shows the time-domain waveforms of the 16 channels and the arrival times selected for each channel, while the right panel shows the spectrogram of Channel 16 for the same signal. The snapping shrimp snap appears as an impulsive broadband signal and exhibits a sequential shift in arrival time across the channels.

A semi-automated procedure was used to extract the arrival times from the experimental snapping shrimp signals. First, candidate snap events were visually screened to confirm that a consistent impulsive arrival structure was observable across all 16 channels. Among the 221 initially identified candidate events, five events exhibiting abrupt channel-to-channel discontinuities in the array-wide TDOA structure were excluded as potentially unreliable arrival-time extractions, leaving 216 events for the final analysis.

For each retained snap event, the Hilbert transform was applied to the signal at each channel to obtain the analytic signal, and the corresponding envelope magnitude was calculated. The arrival time at each hydrophone was then determined automatically as the time of the maximum envelope amplitude. The envelope maximum was used only for events that passed the array-wide continuity screening and was therefore adopted as the experimental arrival-time marker. This procedure does not assume that the maximum envelope peak universally corresponds to the direct path under all multipath conditions. Using the 16 extracted arrival times, the 15-component TDOA vector defined in Equation (8) was constructed relative to Channel 16. The resulting TDOA vector has the same format as the TDOA structures used for training and was subsequently applied to the TDOA-MLP, template matching, SFK, and FDMFP.

In the experimental data, arrival-time estimation may still be affected by multipath propagation, background noise, overlap with other snapping signals, and channel-dependent differences in signal-to-noise ratio. Therefore, the event-screening step was used to restrict the analysis to snaps exhibiting clear and consistent impulsive arrivals across the array. The sensitivity of the proposed method to controlled arrival-time perturbations is examined separately in Section 6.1.

Because the ground-truth source ranges were unavailable, the experimental results were evaluated primarily in terms of consistency among the TDOA-MLP, template matching, SFK, and FDMFP.

## 6. Results

This section presents the source-range and array-tilt estimation results of the proposed TDOA-MLP. First, its prediction performance for source range and array tilt was evaluated using the holdout simulations. The trained model was then applied to the experimental SAVEX15 data, and its results were compared with those obtained using template matching, SFK, and FDMFP.

### 6.1. Simulation Results

In the simulations, the prediction performance of the TDOA-MLP was evaluated using the BELLHOP-based TDOA replica dataset.

**Figure 7:**
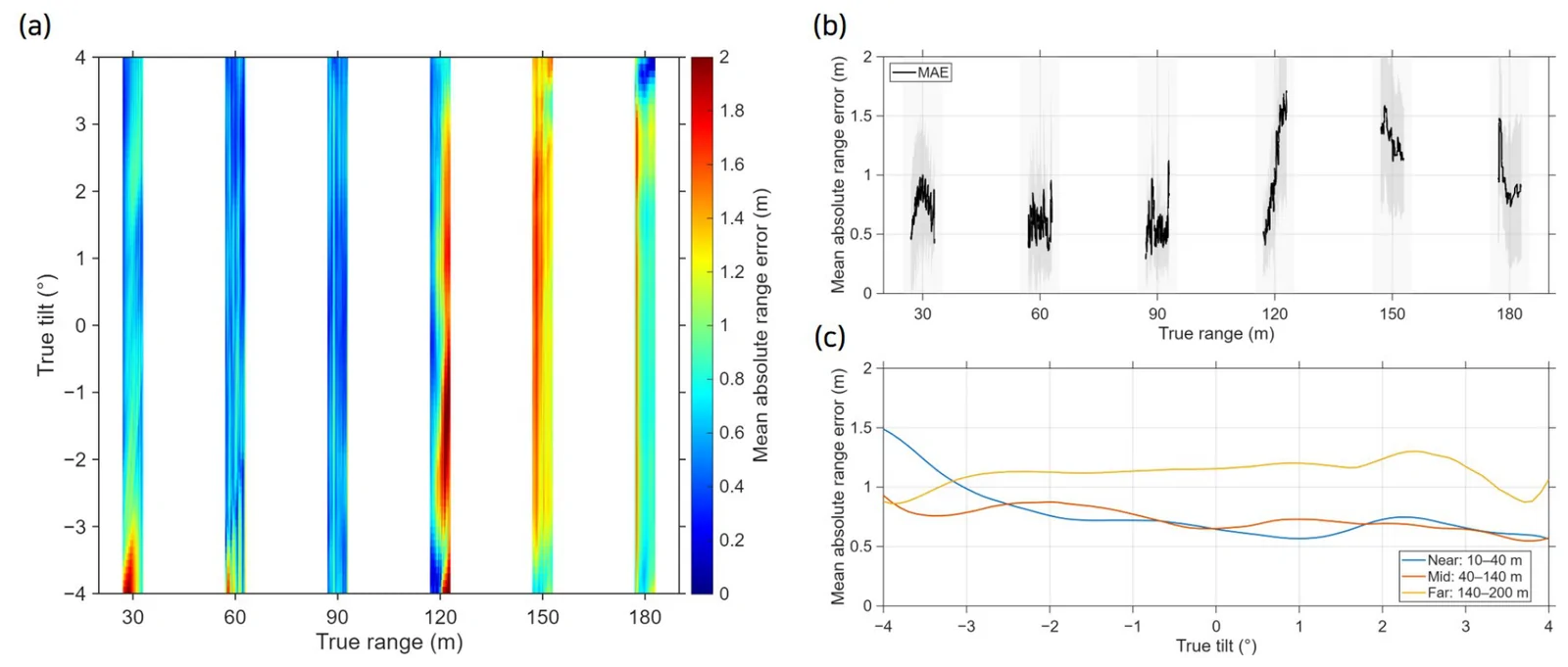
Range-estimation performance of the TDOA-MLP in the blocked-holdout simulation: (**a**) mean absolute range-error map over true range and array tilt, (**b**) range-dependent mean absolute range error, where the black line represents the MAE and the gray shaded region indicates ±1 standard deviation, and (**c**) tilt-dependent mean absolute range error for the near-range (10–40 m), mid-range (40–140 m), and far-range (140–200 m) regions. The evaluation intervals were centered at 30, 60, 90, 120, 150, and 180 m and were separated from the nearest training ranges by at least approximately 2 m.

Figure 7 summarizes the range-estimation performance of the TDOA-MLP under the blocked-holdout evaluation. Unlike the previous point-wise holdout configuration, the present evaluation uses continuous range intervals that were completely excluded from training. The evaluation samples were taken only from the inner portions of the excluded blocks, so that the nearest training ranges were separated from the evaluation ranges by at least approximately 2 m. Across all blocked-holdout samples, the overall mean absolute error (MAE) and root-mean-square error (RMSE) of the estimated range were 0.864 and 1.056 m, respectively. The mean signed error was approximately −0.007 m, indicating little overall systematic bias within the modeled parameter space.

Figure 7a shows the mean absolute range error as a function of true range and array tilt for the blocked-holdout evaluation intervals. The estimation error varies with both source range and array tilt, and relatively larger errors are observed in the far-range evaluation blocks. The error pattern is not perfectly symmetric with respect to the tilt direction. Because the snapping shrimp source is located very close to the seafloor, reversing the sign of the array tilt does not produce a geometrically symmetric source–receiver configuration. Changes in tilt modify the depths and relative positions of the individual hydrophones with respect to the source and seafloor, which in turn affect the direct-path arrival-time structure.

Figure 7b presents the mean absolute range error as a function of true range, with the shaded region indicating ±1 standard deviation of the absolute error. The range-dependent error remains relatively low over the near- and mid-range evaluation blocks but becomes larger and more variable in the far-range region. This behavior is consistent with the reduction in range-dependent TDOA variation as the received wavefront approaches a plane wave at longer source ranges.

Figure 7c shows the mean absolute range error as a function of true tilt for the near-, mid-, and far-range regions. The region-averaged range MAEs were 0.762, 0.728, and 1.119 m for the near-, mid-, and far-range regions, respectively, while the corresponding RMSEs were 0.913, 0.930, and 1.276 m. Thus, the near- and mid-range regions exhibited comparable estimation errors, whereas the far-range region showed a noticeably larger error. The corresponding mean signed errors were 0.525, −0.086, and −0.154 m, respectively. These results indicate that the dominant degradation occurs in the far-range region rather than as a strictly monotonic increase from the near- to the mid- and far-range regions.

Overall, the blocked-holdout results show that the TDOA-MLP can estimate source range for continuous range intervals that were not included in training and were separated from the nearest training samples by approximately 2 m. However, because both the training and evaluation data were generated using the same BELLHOP propagation model and environmental parameterization, these results should be interpreted as generalization within the modeled parameter space rather than as validation under an independent ocean environment.

To further examine the effect of arrival-time uncertainty on the TDOA-MLP estimates, a controlled perturbation analysis was performed using the blocked-holdout test data. For each test sample, independent zero-mean Gaussian perturbations with a standard deviation of $\sigma_{t}$ were added to the arrival times of all 16 hydrophones. Because Channel 16 was used as the TDOA reference, the perturbed TDOA for the *i*-th channel was reconstructed as $\Delta t_{i}^{\prime }=\Delta t_{i}+e_{i}-e_{16}$, where $e_{i}$ and $e_{16}$ denote the perturbations applied to Channels *i* and 16, respectively. The pretrained TDOA-MLP was then applied without retraining. Perturbation standard deviations of 0, 1, 2.5, 5, 10, 25, 50, 75, 100, 150, and 200 µs were considered, and 20 Monte Carlo realizations were evaluated for each nonzero perturbation level.

**Figure 8:**
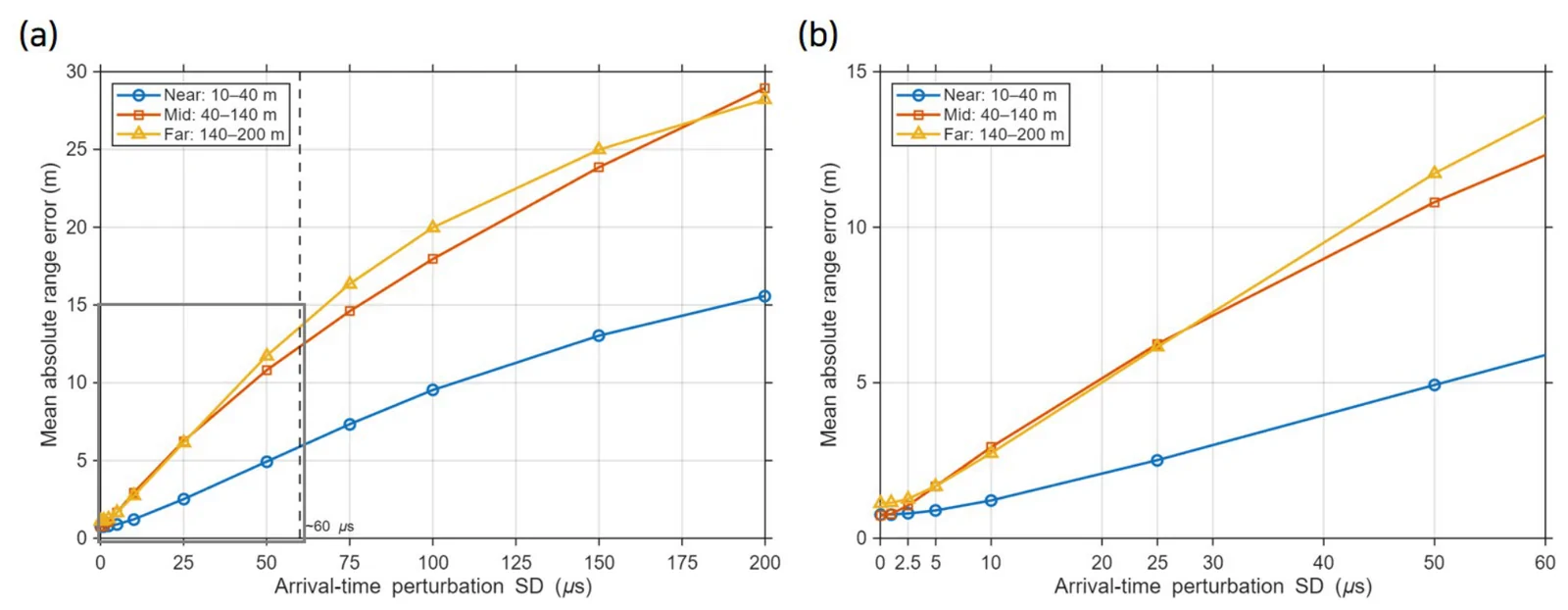
Sensitivity of the TDOA-MLP range estimates to controlled arrival-time perturbations: (**a**) enlarged view for perturbation standard deviations from 0 to 60 µs, where the gray box highlights the 0–60 µs perturbation range and the dashed vertical line indicates approximately 60 µs, corresponding to the characteristic duration of the measured snapping-shrimp pulse, and (**b**) results over the full range from 0 to 200 µs. Independent Gaussian perturbations were applied to the arrival times of the 16 hydrophones, and the 15-dimensional TDOA vectors were reconstructed relative to Channel 16 before inference with the pretrained TDOA-MLP.

Figure 8 shows the resulting mean absolute range errors for the near-, mid-, and far-range regions. With no perturbation, the overall range MAE was 0.864 m. At $\sigma_{t}$ = 1 µs, the MAE increased only slightly to approximately 0.901 m, and at 2.5 µs it increased to approximately 1.081 m. More noticeable degradation occurred as the perturbation level increased further: the overall MAE was approximately 1.544 m at 5 µs and 2.580 m at 10 µs. For larger perturbations, the error increased substantially, reaching approximately 5.592 m at 25 µs and 10.136 m at 50 µs.

The sensitivity also depended on source range. At small perturbation levels, the near-range estimates were affected less than those in the mid- and far-range regions. For example, at $\sigma_{t}$ = 5 µs, the regional MAEs were approximately 0.894, 1.672, and 1.676 m for the near-, mid-, and far-range regions, respectively. At $\sigma_{t}$ = 10 µs, the corresponding MAEs increased to approximately 1.215, 2.930, and 2.737 m. Thus, the mid- and far-range estimates showed a greater sensitivity to arrival-time perturbations than the near-range estimates, which is consistent with the reduced range-dependent variation of the TDOA structure at longer source ranges.

The approximately 60 µs temporal scale indicated in Figure 8a is not intended to represent the actual standard deviation of the arrival-time-picking error in the experimental data. Rather, it corresponds approximately to the characteristic duration of the dominant impulsive portion observed in a representative snapping-shrimp waveform and is included only as a practical reference for interpreting the perturbation range. Therefore, the perturbation results should be interpreted as a controlled sensitivity analysis rather than as an estimate of the actual timing uncertainty in the SAVEX15 measurements.

Overall, the results indicate that the TDOA-MLP estimates are only weakly affected by very small arrival-time perturbations, whereas the range-estimation error increases nonlinearly as the perturbation magnitude reaches several to tens of microseconds. This analysis quantitatively demonstrates the dependence of the proposed method on arrival-time estimation accuracy and highlights the importance of reliable arrival extraction when applying the method to experimental data.

### 6.2. Real Data and Template Matching

For the experimental-data application, the TDOA vectors extracted from snapping shrimp signals received by the SAVEX15 VLA were input to the trained TDOA-MLP to estimate the source range and array tilt. Because the exact ground-truth ranges of the snapping shrimp sources were unavailable, the TDOA-MLP estimates were compared with those obtained using replica-search-based template matching to assess their consistency for the experimental data. Template matching directly searches the same BELLHOP-based TDOA replica dataset and selects the replica condition closest to the input TDOA vector; therefore, it was used as a reference baseline for interpreting the TDOA-MLP results.

**Figure 9:**
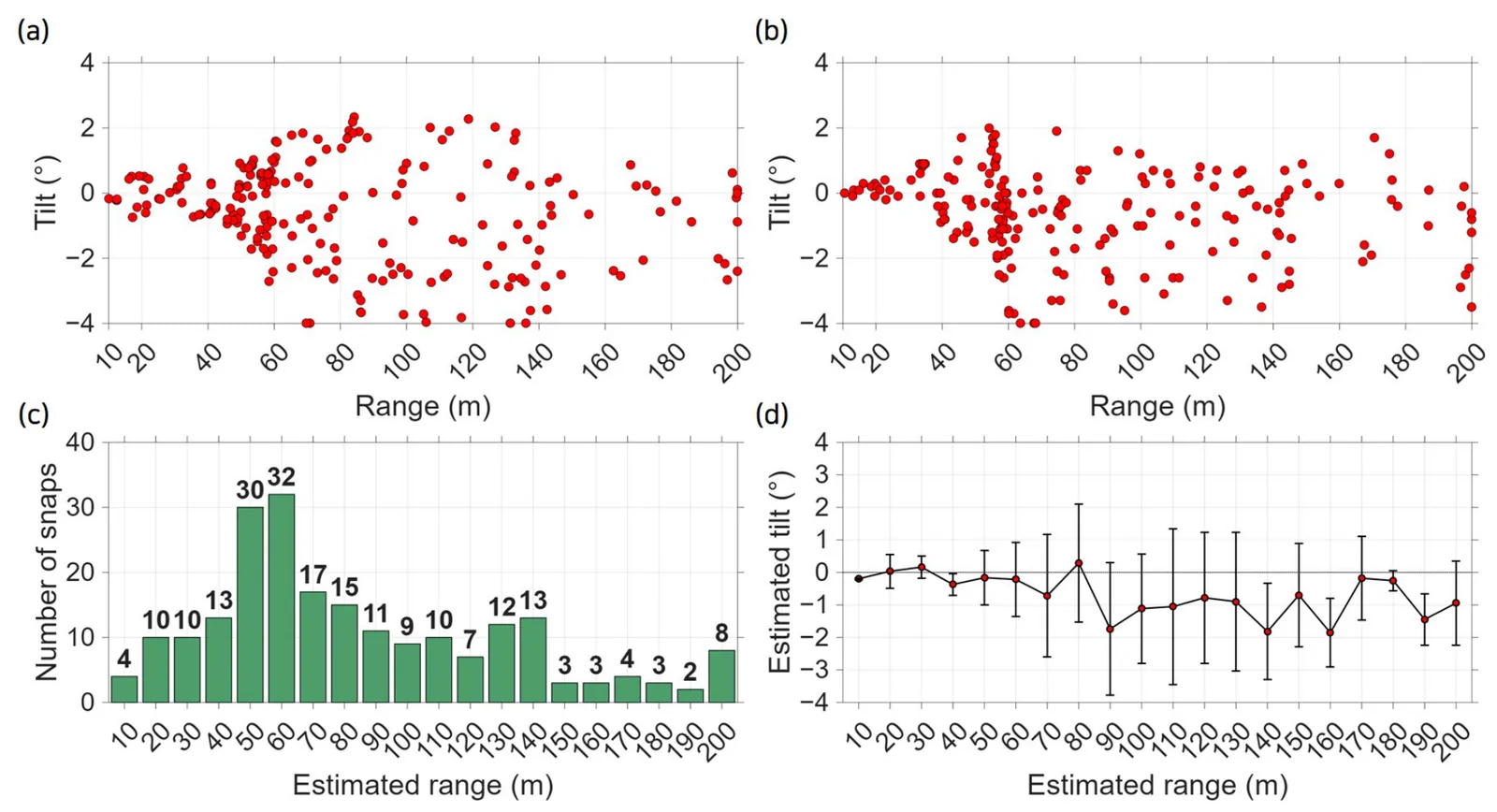
Range–tilt distributions estimated from real snapping shrimp data using (**a**) TDOA-MLP and (**b**) template matching. Panels (**c**,**d**) summarize the TDOA-MLP results using 10-m range bins: (**c**) number of snap events in each estimated-range bin and (**d**) mean estimated tilt with one standard deviation. Each range bin is centered at the labeled value; for example, the 10-m bin includes estimates from 5 to 15 m, subject to the 10–200 m estimation range.

Figure 9 is intended to summarize the overall distribution of the experimental estimates rather than to provide an independent measure of localization accuracy, because ground-truth source positions are unavailable. Figure 9a,b show the range–tilt distributions estimated from the real snapping shrimp data using TDOA-MLP and template matching, respectively. In both cases, a relatively large number of snap events were distributed in the approximately 40–80 m range region, while some estimates extended beyond 100 m. Although differences are observed for individual events, the two methods exhibit generally similar overall range distributions and primary concentration regions.

The tilt estimated from the experimental data should not be interpreted as indicating that the physical orientation of the VLA changed substantially from snap to snap during the approximately 5-min analysis interval. The absolute orientation of the VLA was likely to have remained relatively stable over such a short period. However, because individual snapping shrimp signals originated from different positions and azimuths around the VLA, even a fixed three-dimensional array tilt can produce different apparent tilts when projected onto the vertical plane containing the source and the VLA. A previous study using the same VLA during SAVEX15 similarly interpreted the estimated tilt as the projection of the array orientation onto the source–receiver plane [29]. In addition, differences between the local bathymetry and acoustic environment at the actual source locations and those assumed in the replica model may be partly reflected in the estimated tilt. Therefore, the tilt estimates in Figure 9 are more appropriately interpreted as an effective tilt parameter reflecting both source geometry and environmental mismatch rather than as direct temporal variations in the physical orientation of the VLA.

Differences between the two methods are more apparent for some individual tilt estimates. Template matching selects the nearest condition from the discrete replica grid, whereas TDOA-MLP produces continuous regression outputs through a learned nonlinear mapping. Consequently, the two approaches may respond differently to arrival-time uncertainty, environmental mismatch, and replica-grid discretization even when the same TDOA vector is used as input. The sensitivity of TDOA-MLP to controlled arrival-time perturbations was quantified separately in Figure 8. Some experimental estimates are also located near the boundaries of the prescribed range or tilt domains. Such boundary estimates may reflect input TDOA structures close to the edge of the modeled domain or mismatch between the experimental and replica environments and do not necessarily indicate that the actual sources were located outside the prescribed search region.

Figure 9c shows the number of snap events in each 10-m estimated-range bin based on the TDOA-MLP results. The estimated events span a broad portion of the 10–200 m range domain, with relatively large numbers occurring between approximately 40 and 80 m. In particular, the bins centered at 50 and 60 m contain the largest numbers of events, whereas relatively fewer snaps are observed in the far-range bins beyond approximately 150 m. These counts represent the distribution of estimated ranges for the selected snap events and should not be interpreted directly as the spatial density or actual source distribution of snapping shrimp. Range-dependent detectability, propagation loss, and the event-selection procedure may all influence the number of detected snaps in each range bin.

Figure 9d shows the mean estimated tilt and one standard deviation for the snap events within each estimated-range bin. In the shorter-range bins, the mean tilt remains relatively close to $0^{\circ }$, whereas several mid- and far-range bins exhibit shifts toward negative tilt together with increased variability. Because some far-range bins contain relatively few snap events, their mean and standard deviation can be strongly influenced by a small number of estimates. Accordingly, the range-dependent tilt statistics should be interpreted as variations in the effective tilt parameter associated with source geometry and possible environmental mismatch, rather than as evidence of systematic physical changes in the VLA tilt with source range.

**Figure 10:**
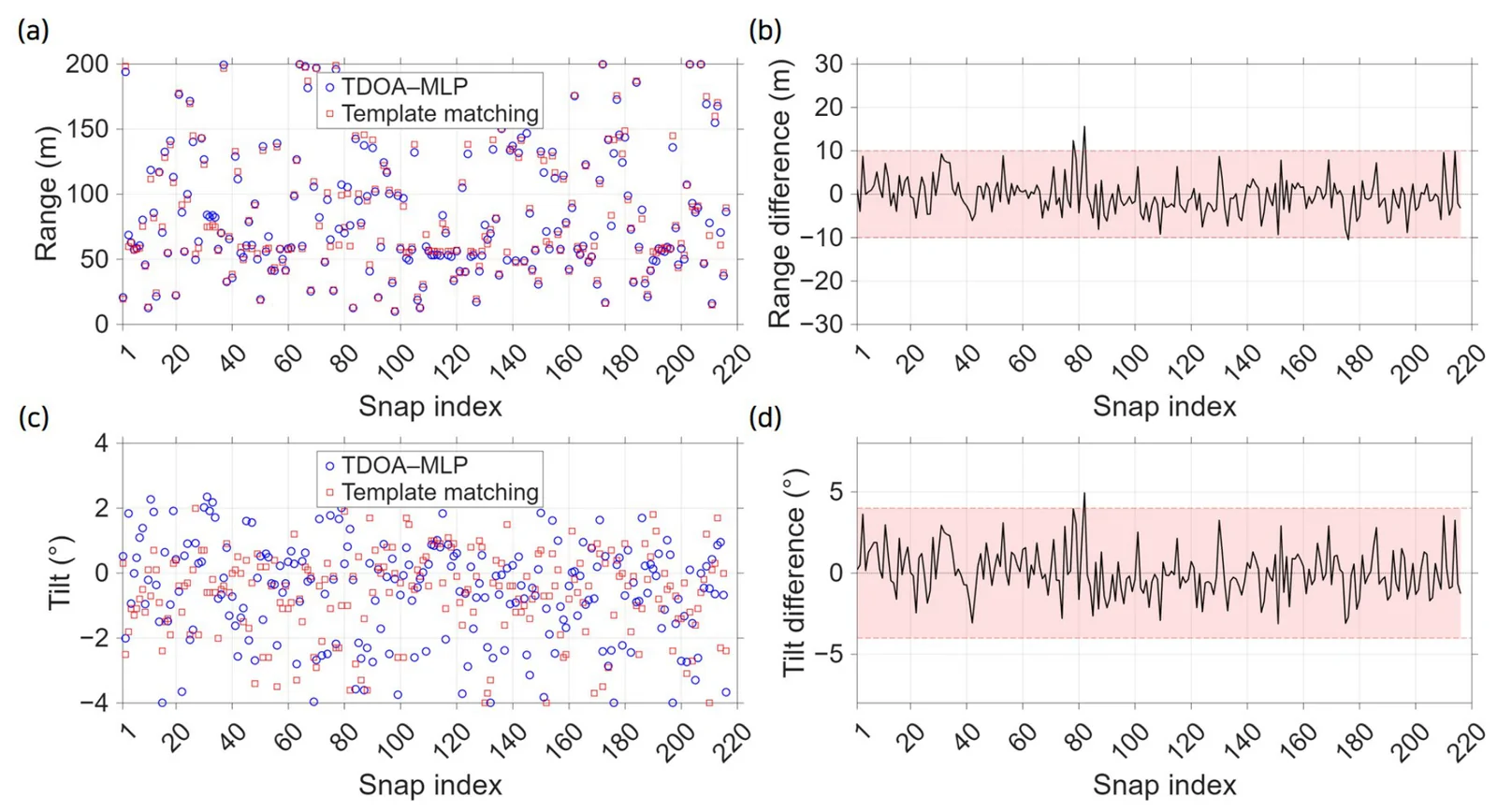
Comparison between TDOA-MLP and template matching for real snapping-shrimp data: (**a**) estimated range, (**b**) signed range difference, (**c**) estimated array tilt, and (**d**) signed tilt difference as functions of snap index. The differences are defined as the TDOA-MLP estimates minus the template-matching estimates. The shaded regions and dashed red lines in (**b**,**d**) indicate the ±10 m and $\pm 4^{\circ }$ difference intervals, respectively.

Figure 10 compares the source range and array tilt estimates obtained using TDOA-MLP and template matching for the real snapping shrimp data as functions of snap index. Figure 10a shows the estimated ranges from the two methods. Although differences are observed for individual events, the two methods generally exhibit similar snap-to-snap range variations over the analysis interval. Figure 10b shows the signed range difference, defined as the TDOA-MLP estimate minus the corresponding template-matching estimate. Most of the differences lie within the ±10 m interval, although larger discrepancies occur for a limited number of snap events.

Figure 10c compares the array tilt estimates obtained using the two methods. The tilt estimates exhibit greater snap-to-snap variability and lower agreement between the methods than the corresponding range estimates. As shown in Figure 10d, most signed tilt differences remain within the $\pm 4^{\circ }$ interval, while several events exhibit larger differences.

Because independent ground-truth measurements of the source range and array orientation were not available for these snapping shrimp events, the experimental comparison does not establish which method provides the more accurate estimate. In particular, the estimated tilt should not be interpreted as a direct measurement of rapid snap-to-snap changes in the physical orientation of the VLA. As discussed for Figure 9, variations in source azimuth can produce different apparent tilts through projection of the three-dimensional array orientation onto the source–receiver vertical plane. Differences in local bathymetry and other environmental conditions, as well as arrival-time uncertainty, may also contribute to variations in the estimated tilt. The differences between TDOA-MLP and template matching therefore reflect not only the uncertainties in the experimental data but also their different estimation mechanisms: continuous nonlinear regression for TDOA-MLP and discrete nearest-replica search for template matching. Accordingly, Figure 10c,d should be interpreted as a comparison of the effective tilt parameters estimated by the two methods rather than as a validation of their absolute tilt accuracy.

### 6.3. Comparison with SFK and FDMFP

To compare the TDOA-MLP results for the experimental data with those obtained using existing physics-based range-estimation methods, the estimates were analyzed together with the SFK and FDMFP results. SFK and FDMFP calculate the response at each candidate range using frequency–wavenumber processing and frequency-difference matched field processing, respectively, and estimate the source location corresponding to the maximum response. In contrast, the TDOA-MLP uses the 15-dimensional TDOA vector extracted from each experimental snap signal as input and jointly estimates the source range and array tilt.

**Figure 11:**
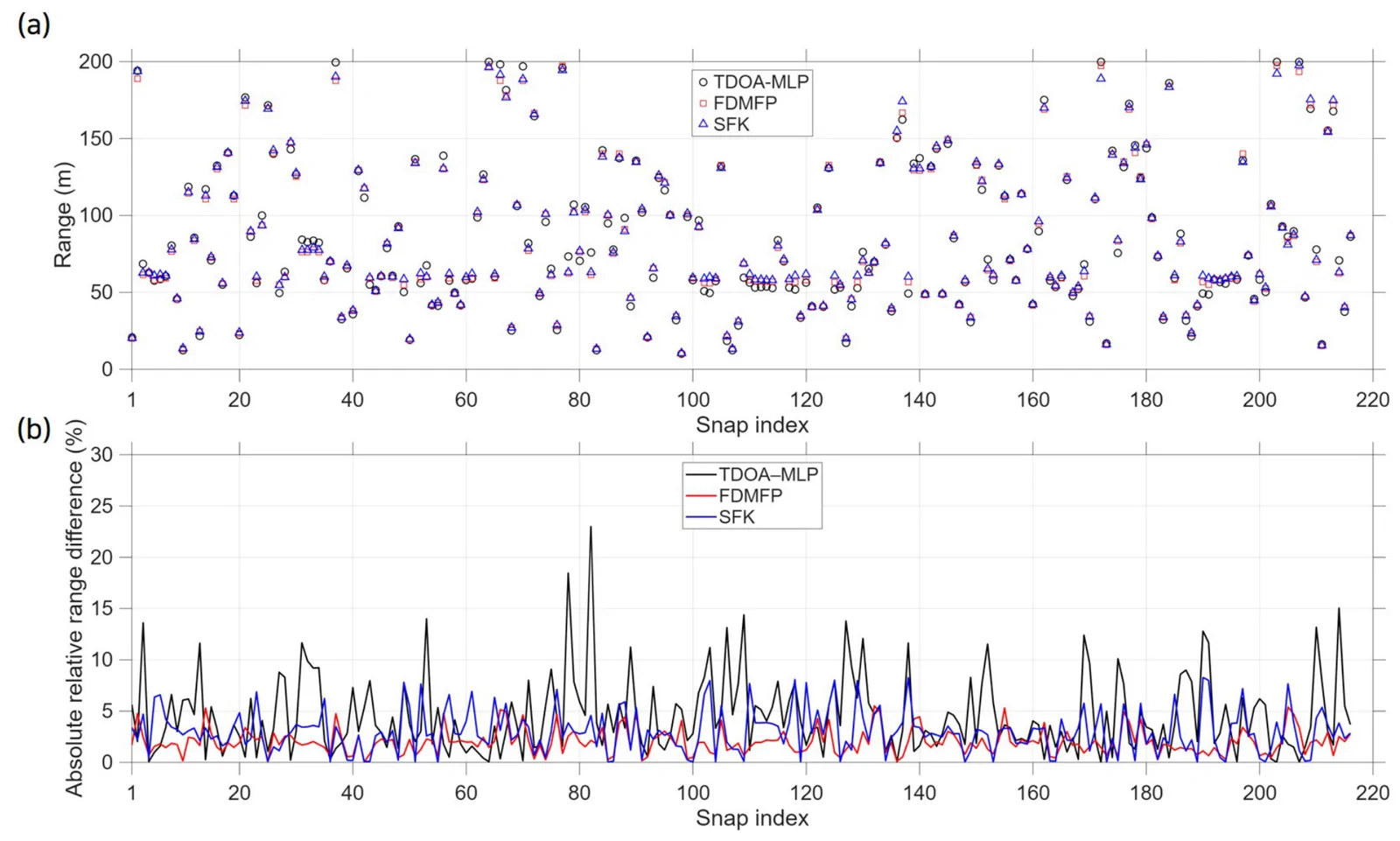
Comparison of real-data range estimates obtained using TDOA-MLP, FDMFP, and SFK: (**a**) estimated range for each snap event and (**b**) absolute relative range differences with respect to the template-matching estimates.

Figure 11 compares the range estimates obtained using TDOA-MLP, FDMFP, and SFK for the experimental snapping shrimp events. Figure 11a shows the estimated ranges from the three methods as a function of snap index. Although differences are observed for some individual events, the three methods generally exhibit similar range distributions and snap-to-snap variation patterns over the analysis interval.

Figure 11b presents the absolute relative range differences of the TDOA-MLP, FDMFP, and SFK estimates with respect to template matching. For the *i*-th snap, the absolute relative range difference for method *q* is defined as follows:

(18) $$D_{q}\left(i\right)=100\left|\frac{{\hat{r}}_{q}\left(i\right)-{\hat{r}}_{\mathrm{TM}}\left(i\right)}{\left[{\hat{r}}_{q}\left(i\right)+{\hat{r}}_{\mathrm{TM}}\left(i\right)\right]/2}\right|,\;q\in \left\{\mathrm{TDOA}\text{-}\mathrm{MLP},\mathrm{FDMFP},\mathrm{SFK}\right\},$$

where ${\hat{r}}_{q}\left(i\right)$ denotes the range estimated by method *q*, and ${\hat{r}}_{\mathrm{TM}}\left(i\right)$ denotes the corresponding template-matching estimate. A smaller value of $D_{q}\left(i\right)$ indicates closer agreement with the template-matching result.

The mean absolute relative range differences were 4.54%, 2.01%, and 3.03% for TDOA-MLP, FDMFP, and SFK, respectively. The corresponding median values were 3.45%, 1.88%, and 2.81%, while the 90th-percentile values were 9.63%, 4.11%, and 6.34%, respectively. Although larger differences are observed for some individual TDOA-MLP estimates, most of the TDOA-MLP results remain within approximately 10% of the corresponding template-matching estimates. Because template matching is not an independent ground-truth reference, these quantities should be interpreted as measures of consistency relative to the common TDOA-based baseline rather than as absolute localization errors.

For the direct comparison between TDOA-MLP and template matching, the mean signed range difference was −0.371 m, while the mean absolute range difference and RMSE were 3.184 and 4.156 m, respectively. These values indicate that the TDOA-MLP estimates did not exhibit a large systematic offset from the template-matching estimates, although event-to-event differences remained present.

The differences among the methods can be related to the distinct input information and estimation principles that they employ. TDOA-MLP and template matching use the interchannel arrival-time structure, whereas SFK and FDMFP rely on the spatial response in the frequency–wavenumber domain and the matching of the frequency-difference acoustic field, respectively. Consequently, the methods may exhibit different sensitivities to arrival-time uncertainty, multipath propagation, environmental mismatch, and discretization of the replica search space. The overall similarity in their snap-to-snap range variations therefore indicates that the TDOA-based estimates are broadly consistent with those obtained using the two physics-based comparison methods, while the differences for individual events reflect the distinct processing principles of the respective approaches.

## 7. Discussion

### 7.1. Physical Interpretation of TDOA-Based Learning

The array-wide TDOA structure reflects the relative path-length differences among the sensors and therefore represents the propagation geometry between the source and the array without depending on the absolute source emission time. Because it does not directly use absolute sound pressure or the frequency-domain acoustic field as input, it can be regarded as a physically interpretable and low-dimensional feature for short-duration snapping shrimp signals.

When the source is located in the near- or mid-range region of the array, the received wavefront exhibits range-dependent curvature rather than behaving as an ideal plane wave. As the source range changes, the relative path lengths to the individual sensors also change, resulting in corresponding variations in the TDOA pattern across the array. Thus, the 15-dimensional TDOA structure contains information related to wavefront curvature and relative path-length differences associated with changes in source range. At longer ranges, the received wavefront approaches a plane wave, and the TDOA variation caused by changes in range becomes smaller. This reduced range sensitivity is consistent with the larger estimation errors observed in the far-range region of the blocked-holdout evaluation in Figure 7.

Array tilt also affects the TDOA structure. When the VLA is tilted, the horizontal and vertical positions of the sensors change, causing variations in the sensor-specific path lengths and arrival times even for the same source range. Accordingly, the TDOA vector contains the combined effects of source range and array tilt. However, the effects of these two parameters are not completely independent, and under some conditions, different combinations of range and tilt may produce similar TDOA structures. This range–tilt coupling may be one reason why the tilt estimates exhibit greater variability than the range estimates in the experimental data.

The blocked-holdout simulation results indicate that a learnable nonlinear mapping exists between the BELLHOP-based TDOA structures and the corresponding range–tilt conditions. In the revised evaluation, continuous source-range intervals were excluded entirely from training, and the evaluation ranges were separated from the nearest training samples by approximately 2 m. The resulting overall range MAE and RMSE were 0.864 and 1.056 m, respectively, with a mean signed error close to zero. These results show that the model can recover range information for continuous regions of the modeled parameter space that were not represented by immediately adjacent training samples. The near- and mid-range regions showed comparable errors, whereas the far-range region exhibited a larger MAE, consistent with the reduced range-dependent variation of the TDOA structure at longer ranges.

However, the blocked-holdout test does not constitute validation under an independent propagation environment. Both the training and evaluation data were generated using the same BELLHOP model, array geometry, and environmental parameterization. Therefore, the blocked-holdout results should be interpreted as evidence of generalization across excluded range intervals within the modeled parameter space, rather than as direct evidence of robustness to environmental mismatch.

In the experimental SAVEX15 data, the TDOA-MLP and template matching exhibited generally similar range distributions and snap-to-snap variation patterns, although differences were observed for individual events. Both methods use the same BELLHOP-based TDOA manifold, but template matching selects the nearest discrete replica whereas the TDOA-MLP applies a learned continuous nonlinear mapping. The mean signed range difference between the two methods was −0.371 m, while the mean absolute difference and RMSE were 3.184 and 4.156 m, respectively. These results indicate that the two methods provide broadly consistent range estimates without a large systematic offset, while still exhibiting event-dependent differences.

The differences between the simulation and experimental results can be partly attributed to uncertainties that are absent from the idealized replica data. In the simulations, the TDOAs share the same propagation model, array geometry, and arrival-time definition as the training data. In contrast, the experimental data may include environmental mismatch, sensor-position uncertainty, multipath variability, and arrival-time estimation error. The controlled perturbation analysis in Figure 8 further showed that the range-estimation error increases as the uncertainty in the channel arrival times increases, with the mid- and far-range regions being more sensitive than the near-range region. This confirms that reliable arrival-time extraction is an important requirement for applying the TDOA-MLP to experimental data.

The larger variability observed in the experimental tilt estimates may also reflect the coupled nature of range and tilt in the TDOA structure. Different range–tilt combinations can produce similar TDOA vectors, and the apparent tilt can vary with the source azimuth even when the physical orientation of the three-dimensional VLA remains nearly unchanged. Under such ambiguous conditions, template matching selects the nearest discrete grid point, whereas TDOA-MLP produces a continuous regression output. Therefore, the estimated tilt is more appropriately interpreted as an effective parameter that reflects the projection of the array orientation onto the source–receiver plane together with possible environmental mismatch, rather than as a direct measurement of the instantaneous physical tilt of the VLA.

The use of the array-wide TDOA structure in the present study is consistent with established TDOA-based localization principles, in which relative arrival times reflect the source–array propagation geometry and can provide information on source location. In the present application, the TDOA-MLP exploits this established geometric information in a low-dimensional form and learns its nonlinear relationship with source range and array tilt from the BELLHOP-based replica dataset.

The generally similar range trends obtained using TDOA-MLP, SFK, and FDMFP show that the range estimates derived from the TDOA representation are broadly consistent with those obtained using physics-based processing methods that rely on different acoustic features. SFK and FDMFP use the spatial response in the frequency–wavenumber domain and the matching of the frequency-difference acoustic field, respectively, whereas TDOA-MLP uses only the relative temporal structure of the selected arrivals. Accordingly, the role of the MLP in this study is not to establish the physical significance of TDOA itself, but to learn the mapping between the BELLHOP-based TDOA manifold and the corresponding range–tilt conditions for snapping shrimp signals received on the SAVEX15 VLA.

More specifically, the TDOA-MLP can be interpreted as learning the nonlinear mapping between the physics-based TDOA manifold formed by the BELLHOP arrival-time replicas and the corresponding range–tilt conditions. This learned mapping is subsequently applied to TDOA vectors extracted from experimental snaps to estimate source range and array tilt.

### 7.2. Range-Dependent Error Characteristics and Propagation-Model Sensitivity

**Figure 12:**
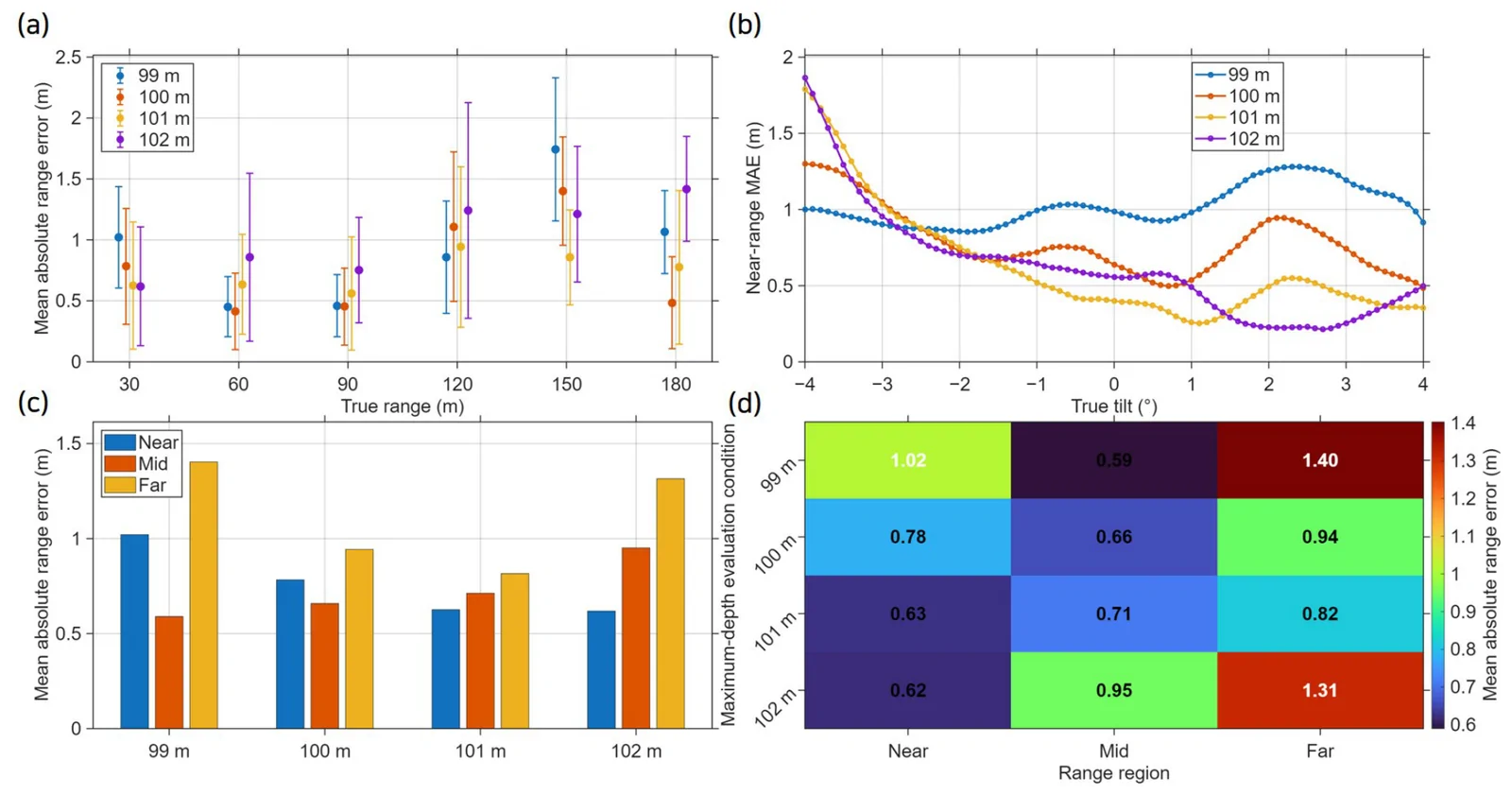
Depth-dependent range-estimation performance of the jointly trained TDOA-MLP under the blocked-holdout evaluation: (**a**) block-wise mean absolute range error with one standard deviation for the six evaluation intervals centered at 30, 60, 90, 120, 150, and 180 m, (**b**) near-range mean absolute range error as a function of true tilt, (**c**) mean absolute range error in the near-range (10–40 m), mid-range (40–140 m), and far-range (140–200 m) regions for each maximum-depth condition, and (**d**) the corresponding regional error map. The same TDOA-MLP, jointly trained using the 99–102 m maximum-depth conditions, was evaluated separately for each maximum-depth condition.

Figure 12 examines the sensitivity of the jointly trained TDOA-MLP to the maximum-depth condition used for evaluation. A single model was trained using the combined replica datasets for maximum depths of 99, 100, 101, and 102 m and was then evaluated separately for each depth condition using the same blocked-holdout range intervals. Thus, the differences shown in Figure 12 reflect depth-dependent variations in the TDOA structure rather than differences among separately trained networks.

Figure 12a summarizes the mean absolute range error for the six blocked evaluation intervals centered at 30, 60, 90, 120, 150, and 180 m. The markers indicate the mean absolute error within each evaluation block, and the vertical error bars indicate one standard deviation of the absolute range error. The depth-dependent performance varies with source range rather than following a single consistent ordering across all evaluation blocks. For example, the 99-m condition exhibits relatively larger errors in the near- and far-range blocks, whereas the 102-m condition shows comparatively low error near 30 m but larger errors in several mid- and far-range blocks. This result indicates that the influence of maximum depth on the learned TDOA mapping is range dependent.

Figure 12b shows the near-range mean absolute range error as a function of true tilt. Under the blocked-holdout configuration, the near-range evaluation corresponds to the 27–33 m interval. The error depends on both tilt and maximum-depth condition, and the relative ordering of the four depth conditions changes across the tilt range. In particular, the 99-m condition remains at approximately 0.9–1.3 m over much of the tilt range, whereas the 101- and 102-m conditions show lower errors for positive tilt values but considerably larger errors near the negative tilt boundary. These results indicate that the sensitivity to maximum depth is coupled with array tilt even within the same short-range evaluation interval.

Figure 12c,d summarize the regional mean absolute range errors for the four maximum-depth conditions. For the 99-, 100-, 101-, and 102-m evaluation conditions, the near-range MAEs were 1.02, 0.78, 0.63, and 0.62 m, respectively. The corresponding mid-range MAEs were 0.59, 0.66, 0.71, and 0.95 m, while the far-range MAEs were 1.40, 0.94, 0.82, and 1.31 m. These results show that no single maximum-depth condition provides the lowest error over all range regions. The 99-m condition gives the lowest mid-range error but the largest near- and far-range errors, whereas the 102-m condition gives the lowest near-range error but exhibits increased error in the mid- and far-range regions. The 101-m condition shows comparatively balanced performance across the three range regions.

The depth-dependent variations can be interpreted as changes in the geometry of the TDOA manifold caused by the environmental parameterization. Changes in maximum depth alter the source depth and the direct-path travel times to the individual hydrophones, thereby modifying the range- and tilt-dependent structure of the TDOA vectors. Because the same jointly trained network is used for all four evaluation conditions, the observed error differences reflect how well the combined learned mapping represents the local TDOA structure associated with each depth condition.

The present results also show that environmental sensitivity cannot be characterized by a simple statement that increasing or decreasing water depth uniformly improves or degrades estimation performance. Instead, the effect depends jointly on source range and array tilt. This dependence becomes especially important in the far-range region, where the TDOA variation with source range is reduced and relatively small environmental changes can produce larger changes in the inferred range. Nevertheless, the present analysis varies only the maximum-depth condition while retaining the same propagation model, sound-speed structure, seabed properties, and array geometry. Therefore, the results should be interpreted as a controlled assessment of sensitivity to one environmental parameter rather than as a complete evaluation of environmental mismatch. The sensitivity to changes in the sound-speed profile was not evaluated separately in the present study and remains an important topic for future validation.

### 7.3. Applicability and Limitations of TDOA-MLP

The results of this study demonstrate that the TDOA-MLP can serve as a low-dimensional learning-based approach for estimating the range of snapping shrimp signals from the array-wide arrival-time structure. The proposed method uses a TDOA vector constructed from a single snap as input and jointly estimates source range and array tilt. The experimental results further show that the range estimates obtained from this TDOA-based representation exhibit trends broadly consistent with those obtained using template matching, SFK, and FDMFP.

However, because the proposed method is trained using BELLHOP-based replicas, its performance may be affected by mismatch between the training and actual environments. Although maximum-depth conditions of 99–102 m were incorporated into a single training dataset, the same jointly trained model exhibited different errors depending on the evaluation environment. Accordingly, practical application requires training data that appropriately represent the sound-speed structure, water depth, array geometry, and source depth of the target environment. In particular, temporal variations in the sound-speed profile may alter the arrival-time structure and should be considered when transferring a trained model to measurements obtained under different oceanographic conditions.

The accuracy of arrival-time extraction directly affects the experimental estimates. Because each TDOA component is formed relative to the reference hydrophone, an error in the arrival time of a single channel can affect one or more elements of the TDOA vector. The controlled perturbation analysis in Figure 8 showed that the range-estimation error remained relatively limited for very small timing perturbations but increased progressively as the perturbation magnitude reached several to tens of microseconds. The mid- and far-range estimates were generally more sensitive than the near-range estimates. These results emphasize the importance of reliable arrival-time extraction and support the use of event-screening or quality-control procedures when applying the method to experimental data.

The blocked-holdout simulations provide a more stringent assessment of range generalization within the modeled parameter space because continuous range intervals were excluded from training and the evaluation samples were separated from the nearest training ranges by approximately 2 m. However, the training and evaluation data were still generated using the same BELLHOP propagation model, sound-speed structure, array geometry, and arrival-time definition. Therefore, the blocked-holdout results do not establish generalization to independent ocean environments.

The additional perturbation analysis quantified the sensitivity of the proposed method to controlled arrival-time uncertainty, and the depth-dependent evaluation in Figure 12 examined its sensitivity to variations in maximum water depth. Nevertheless, further validation is required under broader forms of environmental mismatch, including changes in sound-speed structure, bathymetry, seabed properties, source-height variability, sensor-position uncertainty, and array-geometry errors.

Finally, because the ground-truth source range and array tilt of the experimental snapping shrimp sources were unavailable, the absolute accuracy of the experimental estimates could not be quantified. Comparisons with template matching, SFK, and FDMFP demonstrate consistency among the methods but do not determine which method provides the estimate closest to the actual source location. Controlled experiments using known-position impulsive sources or an independent reference localization system would enable a more rigorous evaluation of the absolute accuracy of the TDOA-MLP.

Overall, the TDOA-MLP can serve as an initial range-estimation or screening approach that simultaneously estimates source range and an effective tilt parameter, provided that the arrival-time structure can be extracted reliably and the mismatch between the training and actual environments remains limited. Future work should extend the training dataset to broader environmental conditions and validate the method using independent ground-truth data and controlled experiments.

## 8. Conclusions

In this study, we proposed a TDOA-MLP method for estimating source range and array tilt from the array-wide TDOA structure of snapping shrimp signals received by a vertical line array. The model was trained using BELLHOP arrival-time replicas generated for the SAVEX15 environment, with source range as the primary output and array tilt as an auxiliary output.

Under the blocked-holdout evaluation, in which continuous source-range intervals were excluded from training, the overall range-estimation MAE and RMSE were 0.864 and 1.056 m, respectively. The regional MAEs were 0.762, 0.728, and 1.119 m for the near-, mid-, and far-range regions, respectively. Additional analyses showed that the estimation performance was affected by arrival-time perturbations and maximum-depth conditions, indicating sensitivity to both timing uncertainty and environmental variability represented in the replica dataset.

For the experimental SAVEX15 snapping shrimp data, the TDOA-MLP range estimates showed overall distributions and snap-to-snap variation patterns generally consistent with those obtained using template matching, SFK, and FDMFP. Relative to template matching, the mean absolute relative range difference of TDOA-MLP was 4.54%, with a median of 3.45% and a 90th-percentile value of 9.63%. Because independent ground-truth source ranges and array orientations were unavailable, these results should be interpreted as demonstrating consistency among the methods rather than absolute localization accuracy. Overall, the TDOA-MLP provides a low-dimensional learning-based approach for estimating the range of impulsive snapping shrimp signals from array-wide TDOA structures. Further validation using broader environmental conditions and independent ground-truth data is required.

## Figures and Tables

**Table 1:** Parameters used to generate the BELLHOP-based TDOA replica dataset.

Parameter	Value
Propagation model	BELLHOP
Number of hydrophones	16
Hydrophone spacing	3.75 m
Reference hydrophone	Bottom hydrophone (Channel 16)
Source type	Snapping shrimp
Source depth	Maximum water depth −0.02 m
Range grid	10–200 m
Range interval	0.01 m
Array-tilt grid	−4°–4°
Tilt interval	0.1°
Maximum water depth	99, 100, 101, and 102 m
Input dimension	15 TDOA components
Output labels	Source range and array tilt

**Table 2:** Architecture of the proposed TDOA-MLP.

Layer	Output Size	Activation	Additional Operation
Input	15	–	Standardized TDOA vector
Hidden layer 1	128	ReLU	Fully connected
Hidden layer 2	128	ReLU	Fully connected
Hidden layer 3	64	ReLU	Fully connected
Output	2	Linear	Source range and array tilt

**Table 3:** Training hyperparameters of the proposed TDOA-MLP.

Parameter	Value
Model	Multilayer Perceptron
Optimizer	Adam
Initial learning rate	$10^{-3}$
$L_{2}$ regularization, α	$10^{-5}$
Batch size	4096
Early stopping iterations	20
Validation fraction	0.10
Tolerance	$10^{-6}$
Data preprocessing	StandardScaler
Training samples	1,500,000
Training-excluded range blocks	25–35, 55–65, 85–95, 115–125, 145–155, 175–185 m
Evaluation range blocks	27–33, 57–63, 87–93, 117–123, 147–153, 177–183 m
Minimum train–test range separation	Approximately 2.01 m
Loss function	Squared error

## Data Availability

The original contributions presented in this study are included in the article. Further inquiries can be directed to the corresponding author.

## References

[1] Johnson M.W., Everest F.A., Young R.W. (1947). The role of snapping shrimp (*Crangon* and *Synalpheus*) in the production of underwater noise in the sea. Biol. Bull..

[2] Everest F.A., Young R.W., Johnson M.W. (1948). Acoustical characteristics of noise produced by snapping shrimp. J. Acoust. Soc. Am..

[3] Cato D.H., Bell M.J. (1992). Ultrasonic Ambient Noise in Australian Shallow Waters at Frequencies up to 200 kHz.

[4] Versluis M., Schmitz B., von der Heydt A., Lohse D. (2000). How snapping shrimp snap: Through cavitating bubbles. Science.

[5] Kim B.-N., Hahn J., Choi B.K., Kim B.-C. (2009). Acoustic characteristics of pure snapping shrimp noise measured under laboratory conditions. Proc. Symp. Ultrason. Electron..

[6] Yuan Z., Richards E.L., Song H.C., Hodgkiss W.S., Yan S. (2018). Calibration of vertical array tilt using snapping shrimp sound. J. Acoust. Soc. Am..

[7] Too Y.M., Chitre M., Barbastathis G., Pallayil V. (2019). Localizing snapping shrimp noise using a small-aperture array. IEEE J. Ocean. Eng..

[8] Choi Y.H., Kim J.S., Byun G. (2023). Source localization based on steered frequency–wavenumber analysis for sparse array. J. Acoust. Soc. Am..

[9] Choi Y.H., Byun G., Kim D., Kim J.S. (2025). Adaptive steered frequency–wavenumber analysis for high-frequency source localization in shallow water. Sensors.

[10] Zhang B., Hu Y., Wang H., Zhuang Z. (2018). Underwater source localization using TDOA and FDOA measurements with unknown propagation speed and sensor parameter errors. IEEE Access.

[11] Liu Y., Wang Y., Chen C. (2023). Efficient underwater acoustical localization method based on TDOA with sensor position errors. J. Mar. Sci. Eng..

[12] Tenorio-Hallé L., Frasier K.E., Frouin-Mouy H., Garrison L.P., Gruden P., Hildebrand J.A., Nosal E.-M., Soldevilla M.S. (2026). A framework for passive acoustic localization and tracking using a wide-baseline array demonstrated with Rice’s whales (*Balaenoptera ricei*). J. Acoust. Soc. Am..

[13] Niu H., Reeves E., Gerstoft P. (2017). Source localization in an ocean waveguide using supervised machine learning. J. Acoust. Soc. Am..

[14] Wang Y., Peng H. (2018). Underwater acoustic source localization using generalized regression neural network. J. Acoust. Soc. Am..

[15] Hu Z., Huang J., Xu P., Nan M., Lou K., Li G. (2021). Underwater acoustic source localization via kernel extreme learning machine. Front. Phys..

[16] Ozanich E., Gerstoft P., Niu H. (2020). A feedforward neural network for direction-of-arrival estimation. J. Acoust. Soc. Am..

[17] Sun D., Fu X., Teng T. (2024). A deep learning localization method for acoustic source via improved input features and network structure. Remote Sens..

[18] Jeong I., Huh H., Jung I.-J., Lee S. (2024). Deep learning-based approach to improve the accuracy of time difference of arrival-based sound source localization. J. Acoust. Soc. Korea.

[19] Yuan P., Wang X., Zhang Z., Zhang J., Zhang H. (2025). Research on underwater acoustic source localization based on typical machine learning algorithms. Appl. Sci..

[20] Worthmann B.M., Song H.C., Dowling D.R. (2015). High frequency source localization in a shallow ocean sound channel using frequency difference matched field processing. J. Acoust. Soc. Am..

[21] Worthmann B.M., Song H.C., Dowling D.R. (2017). Adaptive frequency-difference matched field processing for high frequency source localization in a noisy shallow ocean. J. Acoust. Soc. Am..

[22] Capon J. (1969). High-resolution frequency-wavenumber spectrum analysis. Proc. IEEE.

[23] Hinich M.J. (1981). Frequency–wavenumber array processing. J. Acoust. Soc. Am..

[24] Choi Y.H., Kim D.H., Kim J.S. (2019). Direction finding based on Radon transform in frequency-wavenumber domain with a sparse array. J. Acoust. Soc. Korea.

[25] Pinto F., Kolundžija M., Vetterli M. (2014). Digital acoustics: Processing wave fields in space and time using DSP tools. APSIPA Trans. Signal Inf. Process..

[26] Abadi S.H., Song H.C., Dowling D.R. (2012). Broadband sparse-array blind deconvolution using frequency-difference beamforming. J. Acoust. Soc. Am..

[27] Porter M.B. The Acoustics Toolbox. http://oalib.hlsresearch.com/AcousticsToolbox/.

[28] Byun G., Akins F.H., Gemba K.L., Song H.C., Kuperman W.A. (2020). Multiple constraint matched field processing tolerant to array tilt mismatch. J. Acoust. Soc. Am..

[29] Song H.C., Byun G. (2022). An overview of array invariant for source-range estimation in shallow water. J. Acoust. Soc. Am..

[30] Song H., Cho C., Hodgkiss W., Nam S., Kim S.-M., Kim B.-N. (2018). Underwater sound channel in the northeastern East China Sea. Ocean Eng..

